# Influenza Vaccine Technology Transfer: A Mixed-Methods Study with Vaccine Manufacturers and Global Experts to Assess Successes, Challenges, and Opportunities

**DOI:** 10.3390/vaccines14060522

**Published:** 2026-06-11

**Authors:** Christopher Chadwick, Erin Sparrow, Claudia Nannei, Jessica Taaffe, William Ampofo, Antoine Flahault, Seth Berkley

**Affiliations:** 1 Institute of Global Health, Faculty of Medicine, University of Geneva, 1211 Geneva, Switzerland; 2 World Health Organization, 1211 Geneva, Switzerland; 3 Global Renaissance Enterprises, Washington, DC 20011, USA; jtaaffe@globalrenaissanceenterprises.com; 4 Department of Medical Laboratory Sciences, School of Biomedical and Allied Health Sciences & Virology Department, Noguchi Memorial Institute for Medical Research, College of Health Sciences, University of Ghana, Accra 23321, Ghana; 5 Université Paris Cité, Xavier Bichat Hospital, Inserm, 75018 Paris, France; 6 Pandemic Center, School of Public Health, Brown University, Providence, RI 02912, USA

**Keywords:** technology transfer, influenza vaccine, local production, access to vaccines, epidemic and pandemic preparedness

## Abstract

Background/Objectives: Technology transfer (TT) has been identified as a global health priority due to its impact on improving access to vaccines, including for pandemic influenza preparedness and response through bilateral and multilateral mechanisms. This study aimed to (1) characterize examples of influenza vaccine TT (IVTT) and (2) identify key lessons learned that may inform future activities relevant for next-generation influenza vaccine technologies. Methods: Using a contingent effectiveness model, a convergent mixed-methods study was conducted with vaccine manufacturers and global experts to capture quantitative survey data on IVTT activities and enablers and qualitative data on successes, challenges, and opportunities for IVTT through interviews, complemented by secondary data from peer-reviewed and grey literature to characterize additional IVTT observations. Results: This study included 24 participants, including 14 representatives from 13 vaccine manufacturers and 10 experts. Interviews were conducted with representatives from eight manufacturers and seven experts. Eighteen IVTT observations were identified through the surveys and interviews, of which 15 IVTT transfers were completed and 13 resulted in an approved vaccine. Secondary data provided additional evidence on eight IVTT recipients and one supplier, expanding the range of institutional and programmatic contexts assessed. Shorter IVTT completion and vaccine approval timelines were observed in association with prior TT experience and private management structures for manufacturers, for pre-pandemic/pandemic influenza vaccines versus seasonal influenza vaccines, and among bilateral transfer mechanisms (versus multilateral mechanisms) and fill/finish transfer methods. Manufacturers also described spillover benefits, including the use of IVTT-related know-how for the development of COVID-19 and routine vaccines. Both manufacturers and experts generally agreed on a list of 17 enablers for successful IVTT and ranked government commitment to vaccine production and procurement as the top enabler. Findings from the literature-based observations were consistent with primary data and included additional public sector recipient experiences, evidence of widespread human capital development, and a commentary on the importance of the demand environment. Conclusions: Assessed IVTT activities across primary and secondary data sources yielded commercial and spillover benefits as described in the contingent effectiveness model and provided a triangulated analysis of IVTT experiences across manufacturers, experts, and documented cases. Participants agreed that effective technology transfer is contingent upon a host of determinants. Using a systematic application of the contingent effectiveness model to IVTT, this study provided an exploratory analysis of past activities among vaccine manufacturers and experts. While certain nuances for influenza were identified, the lessons learned from this study may be applicable for other TT activities, including those to support pandemic preparedness. The contingent effectiveness model is a useful tool to inform and evaluate future TT activities.

## 1. Introduction

Due to the complexities, costs, and risks associated with vaccine development, global organizations, governments, and manufacturers promote technology transfer (TT) as a method for increasing access to vaccines, such that production processes, know-how, and technologies are transferred from one entity (the transfer supplier) to another (the transfer recipient). There is broad recognition that TT not only improves access to vaccines through local production but also builds capacity for research and development (R&D) and innovation for developing country vaccine manufacturers (DCVMs) [1,2].

TT can be conducted through a variety of mechanisms, such as bilateral agreements or multilateral partnerships, and can involve the transfer of different vaccine development stages, such as upstream R&D, end-to-end production, or fill/finish of the final product using bulk antigen provided by the transfer supplier. Some TT agreements include a stepwise transfer in which the recipient begins with fill/finish and moves to fully integrated vaccine production once approval and distribution processes are adequately established [3].

When conducting TT, several factors need to be considered, including costs, regulatory processes, and timelines. Challenges in TT have been documented across institutional and national levels, including R&D capacity at the recipient site, clinical trial expertise, quality control experience, human resources, and national regulatory authority (NRA) capacity [3]. Additional challenges have been documented for other technologies that can apply to vaccines: supply constraints of raw materials and timely and costly transfers to new facilities without a TT platform for biologics, difficulties implementing equipment and misalignment of products with local needs for medical equipment in humanitarian settings, and unclear profit opportunities and limited technological understanding for space technologies [4,5,6]. Several enablers for successful TT have been identified across policy, institutional, and regulatory categories. Examples include public policies to support TT through demand generation, training and retention of a highly skilled workforce, and early and sustained engagement with regulators [4,7,8].

A contingent effectiveness model was proposed to examine TT, including who is transferring the technology, how it is being transferred, what is being transferred and to whom, in what environment, and what impacts the TT may have. The model includes five determinants of effectiveness: transfer recipient, transfer supplier, transfer object, transfer media, and demand environment. It also includes six criteria for assessing the effectiveness of TT: out-the-door (i.e., was the technology actually transferred), market impact, economic development, political advantage, development of scientific and technical (S&T) human capital, and opportunity costs [9]. In 2015, the model was revised to include an additional effectiveness criterion on public value, accounting for broader societal outcomes of TT. There is recognition that effectiveness analyses tend to focus more on the out-the-door criterion, which emphasizes transfer completion over use and impact of the technology, and the market impact criterion, which emphasizes commercial success but may ignore spillover benefits important for public sector and non-profit stakeholders [10].

TT activities among DCVMs have been shown to improve access to vaccines for several scenarios: (1) the disease is globally significant but only a small number of manufacturers were producing the relevant vaccine at a limited capacity (e.g., hepatitis B vaccines), (2) the disease is not a priority in high-income countries (HICs) and there was limited interest among manufacturers in HICs to produce the relevant vaccine (e.g., meningitis A vaccines), or (3) the disease is globally significant but global vaccine production capacity was limited (e.g., pandemic influenza vaccines). Previous assessments have characterized TT effectiveness and impact. For example, 92 TT observations were identified between 1988 and 2010, with the majority occurring in India, China, and Brazil and with influenza, hepatitis, and rotavirus as key disease targets [3]; a more in-depth analysis of 73 of those TT observations showed that 29 resulted in a nationally licensed product, of which nine obtained WHO prequalification (PQ) [11]. These analyses documented the heterogeneity of TT, including geographic location of recipients and suppliers, disease targets, the transfer mechanisms and methods. However, no data on the demand environment were presented, and outcomes presented were limited to out-the-door and market impact criteria. During the COVID-19 pandemic, the Clinton Health Access Initiative tracked 112 TT commitments to support local vaccine manufacturing, with commitments in Asia (*n* = 51) far exceeding those in Africa (*n* = 18), Latin America (*n* = 14), and the Middle East (*n* = 9); however, there are no data on outcomes from those TT commitments [12]. Additionally, the Medicines Patent Pool is tracking mRNA vaccine initiatives and, as of May 2025, noted 26 initiatives that have TT as a focus, 20 of which indicated end-to-end vaccine production capability as the goal [13].

The role of TT in epidemic and pandemic preparedness and response has been emphasized at the highest global levels, including most recently in the Pandemic Agreement that was adopted through resolution 78.1 by the Seventy-eighth World Health Assembly (WHA) in May 2025, whereby WHO Member States recognized TT as a mechanism for achieving sustainable and geographically diversified production of vaccines in low- and middle-income countries (LMICs) [14]. This role was previously highlighted in 2005, when in response to the potential pandemic threat of highly pathogenic avian influenza A(H5N1) and concentration of influenza vaccine production capacities in HICs, the Fifty-eighth WHA adopted resolution 58.5 to request the World Health Organization (WHO) work with partners to address the global shortage of influenza vaccines and enable the implementation of influenza vaccination strategies in WHO Member States [15]. Resolution 58.5 was the foundation for the Global Action Plan for Influenza Vaccines (GAP) in 2006, a 10-year strategy which promoted influenza vaccine TT (IVTT) to increase access to influenza vaccines by low- and middle-income countries (LMICs) in the event of an influenza pandemic [16]. To support GAP’s objectives, WHO established the Technology Transfer Initiative (TTI) to transfer egg-based inactivated influenza vaccine (IIV) and live attenuated influenza vaccine (LAIV) production technologies to manufacturers in 14 LMICs [17,18,19]. TTI’s activities were facilitated by an IVTT hub at the Netherlands Vaccine Institute (NVI) [20], an adjuvant TT hub with the University of Lausanne [21], training centers established by the United States Biomedical Advanced Research and Development Authority [22,23], and a roster of experts assembled through a WHO Technical Advisory Group (TAG) [18,24] and PATH [25].

Global health researchers and advocates continue to promote IVTT to increase equitable access to vaccines, including for pandemic influenza [26]. Through the Pandemic Influenza Preparedness (PIP) Framework, WHO Member States recognize the importance of IVTT as a potential benefit for countries in exchange for vaccine researchers, developers, and manufacturers accessing influenza viruses with pandemic potential for the development of medical countermeasures [27]. However, as of March 2026, no vaccine manufacturers had agreed to include TT commitments in benefit-sharing agreements signed with WHO, opting instead for the real-time supply of vaccines in the event of an influenza pandemic [28].

Currently licensed seasonal influenza vaccines include IIV, LAIV, recombinant influenza vaccines (RIV), adjuvanted IIV, and high-dose IIV. Monovalent pre-pandemic and pandemic influenza vaccines have been developed using IIV, LAIV, and RIV technologies. Additionally, next-generation influenza vaccines (NGIVs) across a variety of platforms, including mRNA, as well as combination respiratory vaccines addressing influenza and SARS-CoV-2, are in clinical development, with one combination influenza-SARS-CoV-2 mRNA vaccine receiving marketing authorization in April 2026 [29,30]. Although there has been considerable progress in increasing global influenza vaccine production capacities and expanding production capabilities to LMICs since the start of GAP, production capacities are still largely concentrated in HICs, and the majority still utilize eggs as the production substrate [31].

As NGIVs are developed and the use of enhanced seasonal influenza vaccines (e.g., RIV, adjuvanted IIV) is expanded, it will be important for governments and manufacturers in LMICs to assess how they may access the technologies, and TT may be an efficient mechanism to facilitate access among DCVMs. In this study, we aimed to distill successes and challenges from past IVTT activities that could inform future efforts. Specifically, we sought to:

- Characterize determinants and effectiveness of past IVTT activities;
- Assess the impact of determinants on the effectiveness of IVTT;
- Identify enablers that are considered important for IVTT from the perspectives of vaccine manufacturers and global experts.

## 2. Materials and Methods

### 2.1. Conceptual Framework

The revised contingent effectiveness model served as the conceptual framework for this study [10]. Although the model has been widely cited in the broader TT literature, its use in IVTT research has only appeared in narrative discussions rather than empirical studies involving primary data from TT participants [11]. This study applied the model as a framework to characterize IVTT determinants, effectiveness criteria, and enabling conditions. Table 1 describes the determinant domains and effectiveness criteria, their focus, and application to this study.

### 2.2. Study Design

This retrospective, cross-sectional study utilized a convergent mixed-methods research design to survey and interview vaccine manufacturers and experts that have participated in IVTT. The goal was to obtain both quantitative and qualitative data from every participant and to prioritize each dataset equally. The approach was grounded in a pragmatist worldview, emphasizing the practical consequences of IVTT and the use of complementary data to address the research aims [32]. Relevant IVTT variables were selected based on the study aims, prior TT literature, and the availability of data that could be consistently captured through the survey and interviews. Variables were then mapped deductively to the determinant domains and effectiveness criteria of the contingent effectiveness model.

### 2.3. Study Participants

**Figure 1:**
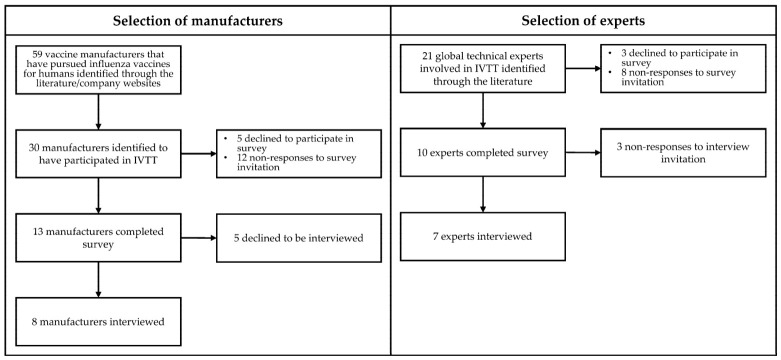
Flow diagram for the identification and selection of study participants.

Inclusion criteria for the study were currently established vaccine manufacturers that have entered into a TT agreement, either as a supplier or recipient, for any influenza vaccine for use in humans. Purposeful sampling was used to select participants representing local/national vaccine manufacturers, multinational corporations (MNCs), and experts. Figure 1 provides a flow diagram for the identification and selection of study participants.

Because public documentation of IVTT participation is limited, eligibility verification required direct outreach to manufacturers and expert networks. Fifty-nine vaccine manufacturers potentially relevant for this study were identified through the literature [18,33,34,35], industry association websites, and individual manufacturer websites and asked by email to confirm study eligibility. Thirty manufacturers were determined to have participated in IVTT, including eight as suppliers and 22 different manufacturers as recipients. In addition to the vaccine manufacturers, 21 experts who supported the WHO TTI, including through the TAG, were contacted to participate in the study [18,20,21,24,25,36]. Five manufacturers declined to participate in the survey, 12 did not respond to the survey invitation, and five declined to be interviewed following completion of the survey. Three experts declined to participate in the survey, eight did not respond to the survey invitation, and three did not respond to interview invitations following completion of the survey.

### 2.4. Data Collection

The survey was designed in English using the LimeSurvey platform (Version 6) to capture information on participant characteristics, determinants and effectiveness of IVTT (limited to manufacturers), and enablers of IVTT. The third section of the survey included a set of questions to assess the importance and order of significance of 17 enablers of vaccine TT identified through the literature [3,37,38]. Importance was assessed on a 5-point Likert scale. Order of significance was assessed through rankings of the top five enablers. Separate surveys were designed for local/national vaccine manufacturers, MNCs, and experts, which are available in the Supplemental Material (Documents S1–S3). The surveys were reviewed by the researchers for clarity and alignment with the contingent effectiveness model. Survey links were shared with participants along with instructions for completing the survey and a fillable Microsoft Word version of the survey to support data collection. For the local/national manufacturers and MNCs, focal points for the survey were identified either through previous production capacity surveys [33] or by staff at the International Federation of Pharmaceutical Manufacturers and Associations, Biotechnology Innovation Organization, and Developing Countries Vaccine Manufacturers Network. Focal points were encouraged to work with their colleagues to provide data at an institutional level. Participants were assured that information collected in the survey would remain confidential and secure, and final data would be anonymized after collection. The survey remained open between March and September 2023. Up to three follow-up emails were sent to identified participants.

Interview guides were designed in English using a semi-structured format with a set of 14 pre-defined questions to capture information on (1) overall experience and expertise with TT, including for influenza; (2) determinants and effectiveness of IVTT; and (3) enablers of IVTT. Separate interview guides were designed for vaccine manufacturers and experts (Documents S4 and S5 in the Supplementary Materials). For those participants that indicated their willingness to be interviewed, interview guides were provided in advance. Interviews were conducted in English using Microsoft Teams between May and October 2023. Survey results were analyzed prior to an interview, and any questions about the survey data were clarified during the interview given its semi-structured design. System-generated transcripts were reviewed against the recordings for validation and correction, as needed. Any questions or issues with the transcripts were addressed with participants. Final interview transcripts were anonymized. If participants were unavailable for an interview, they were offered the opportunity to provide written responses by email using the questions provided in the interview guide.

To complement primary data collected in this study, secondary data on determinants, effectiveness outcomes, and enablers were collected for the remaining IVTT recipients and suppliers that did not participate in the survey. Searches were conducted in English in PubMed, Google Scholar, and relevant grey literature sources, including WHO reports, national government reports, and manufacturer websites. Search terms included combinations of “influenza vaccine,” “technology transfer,” and “local production,” as well as institution-specific queries. Using the conceptual framework, sources were screened for relevance and included if sufficient details were available for coding across the institution and its capabilities, the transfer object and media, the demand environment, and at least one effectiveness criterion. Sources were excluded if they focused on TT for non-influenza or veterinary vaccines or described the IVTT observations only in general terms.

For survey data and interview transcripts, an anonymization log was developed to account for replacement, aggregation, and removal of any identifiable data. Following transcription, audio/video files from the interviews were destroyed. Data collected during the study remained confidential and secure and were only accessible by the research team.

### 2.5. Data Analysis

Survey data were compiled in LimeSurvey and exported to IBM SPSS Statistics (Version 29) for cleaning and analysis. Focal points were contacted if there were missing data or for clarifying questions. Descriptive statistics were generated for all survey data. Regarding overall rankings of TT enablers, a weighted ranking method was applied, whereby for each participant, the highest-ranked enabler was assigned five points through the fifth-ranked enabler that was assigned one point. Weighted scores for each enabler were summed across all participants and ranked in descending order to determine overall importance. This method was selected because participants only ranked their top five enablers, and the weighting accounted for both the frequency with which each enabler was selected and its relative priority among participants.

For IVTT effectiveness, two new variables were derived from the survey data: time to IVTT completion and time to influenza vaccine approval. Time to IVTT completion was defined as the duration (in years) between the formal initiation of the relevant IVTT agreement (i.e., launch of the project) and the documented completion of the agreement (i.e., confirmation that production processes or technology training were finalized). Time to influenza vaccine approval was defined as the duration (in years) between the initiation of the IVTT agreement and the first regulatory approval of the resulting influenza vaccine. When initiation and completion or approval occurred within the same calendar year, the duration was coded as one year to avoid zero-year values.

In addition to descriptive statistics of ordinal data related to the importance and order of significance of IVTT enablers, non-parametric tests were conducted because data were not normally distributed. Mann–Whitney U tests were used when assessing two independent groups (all manufacturers versus experts), and Kruskal–Wallis H tests were used with three independent groups (recipients, suppliers, and experts). Where statistically significant differences were identified in Kruskal–Wallis H tests, post hoc pairwise comparisons were conducted to determine which groups differed. A Bonferroni adjustment was applied to the pairwise comparisons to reduce the risk of type I error from multiple testing. Statistical significance was set at *p* < 0.05 for primary analyses, except for Bonferroni-adjusted post hoc comparisons following Kruskal–Wallis H tests where significance was set at *p* < 0.017 because three pairs were compared.

Anonymized interview transcripts were exported to MAXQDA Analytics Pro 24 for thematic analysis using a structured codebook with separate codes for the five determinant domains, six effectiveness criteria, and 17 enablers. Two coders performed deductive coding on a subset of four transcripts (representing two manufacturers and two experts) to validate the codebook [39]. Inter-rater reliability was assessed in MAXQDA based on agreements and disagreements for code occurrence in the documents and coding of individual segments. For code occurrence, overall agreement for the four transcripts was 83% with individual Cohen’s kappa coefficient values of 0.65, 0.81, 0.94, and 0.94. These values indicated acceptable to strong agreement for code occurrence, supporting the reliability of the structured codebook at the document level. However, for coding of individual segments, the kappa coefficient was 0.47, which indicated weak agreement and further codebook refinement. Disagreements between the coders were documented in a table along with the solutions for reconciliation. Based on the inter-rater reliability results, the structured codebook was revised to include the 17 enablers as subcodes to corresponding determinant domains and clarify inclusion/exclusion criteria for each code (Document S6 in the Supplementary Materials). Following reconciliation and the update to the codebook, overall agreement for code occurrence was 98% with individual kappa coefficient values of 0.93, 1.00, 1.00, and 1.00, and for coding of individual segment, the kappa coefficient was 0.91. Post-reconciliation values indicated strong agreement, suggesting that the revised codebook provided a reliable and consistent basis for coding the remaining transcripts by a single coder. Memos were used for each code to summarize themes and any diverging views and identify representative quotes.

For the secondary data analysis, the compiled literature for each institution were exported to MAXQDA Analytics Pro 24 for thematic analysis using the revised codebook. Memos were used for each institution to summarize IVTT determinants, effectiveness criteria, and enablers.

The quantitative and qualitative analyses were conducted in line with best practices for mixed-methods research, emphasizing complementarity and triangulation across data types to enhance validity and interpretation [32]. Quantitative findings informed patterns and associations among determinants, effectiveness criteria, and enablers, while qualitative analyses provided additional context and evidence of agreement or disagreement. Integration was achieved throughout the study: (1) at the study design level with the use of the adapted contingent effectiveness model to align the survey, interviews, and codebook; (2) at the data collection level where survey data informed the semi-structured interviews; and (3) at the interpretation level where quantitative and qualitative findings were presented in narrative discussions for each determinant domain, effectiveness criteria, and enabler [40].

## 3. Results

A total of 24 individuals completed the survey. Of the 30 manufacturers known to have participated in IVTT as a recipient or supplier, representatives from 13 responded to the survey representing a 43% response rate. This included 13 representatives from 12 local/national vaccine manufacturers and one representative from an MNC. Ten of the 21 experts responded to the survey representing a 48% response rate.

Fifteen interviews were conducted with representatives from eight vaccine manufacturers and seven experts, representing 62% and 70% of the total participating manufacturers and experts, respectively. The interviewed manufacturers included seven recipients and one supplier. All interviews with manufacturers included one representative from the institution, except for one interview which included two representatives. The eight vaccine manufacturer interviews included one that was unable to participate in an interview but did provide written feedback to the interview questions.

The representative from an MNC completed the parts of the survey dedicated to TT enablers and declined to be interviewed. For participant characteristics, this individual is included in the list of 11 experts. However, for the data on enablers, they are identified as a manufacturer for group analyses and as a transfer supplier for sub-group analyses.

The literature review to identify additional IVTT observations not represented in the survey and interviews yielded findings from nine separate institutions not included in the survey. These findings are presented in Section 3.6 and are excluded from Section 3.1, Section 3.2, Section 3.3, Section 3.4 and Section 3.5 that present the primary data from the survey and interviews.

### 3.1. Participant Characteristics

The 12 participating local/national vaccine manufacturers, which are anonymized for the study, are located in Argentina, Bangladesh, Brazil, China, India, Indonesia, Republic of Korea, Serbia, South Africa, Thailand, and Viet Nam. Participants provided details on their management structure, annual installed production capacity for all vaccines, landscape of vaccines that have been approved by an NRA or achieved WHO PQ, status of seasonal influenza vaccine production, role as an IVTT recipient or supplier, and participation in the WHO TTI. Participating manufacturers reflected diversity across geography, management structure, and production scale and product landscape. They were based across five WHO regions, were mostly based in upper MICs, and included both private and public institutions. Annual production capacity was evenly distributed across the four categories, suggesting representation from smaller, medium, and larger vaccine producers. Most manufacturers were current seasonal influenza vaccine producers or had a product in phase 3 clinical trials, and the majority participated in IVTT as recipients. These characteristics are described in Table 2.

Experts provided details on their primary area of expertise as it related to IVTT, if they provided direct technical assistance to a manufacturer for IVTT, and if they were a member of the WHO TAG. Participants reflected substantial familiarity with IVTT as most had served as members of the WHO TAG and almost half provided direct technical assistance to manufacturers. Their areas of expertise spanned manufacturing, product strategy, public health, regulatory issues, and R&D. These characteristics are described in Table 3.

### 3.2. IVTT Observations

Ten recipients and one supplier provided information for 18 unique IVTT observations, ranging from start dates of 1999–2018 and completion/termination dates of 2008–2023; 17 observations were recorded through the survey, and one additional observation was described during an interview. Seven manufacturers provided information for one IVTT observation, while the remaining four provided information for multiple observations, with one recipient having four observations. Document S7 in the Supplementary Materials includes a detailed overview of the 18 observations. Summary findings for the observations are presented in the following sections.

### 3.3. Determinants

#### 3.3.1. Transfer Recipient

The 10 IVTT recipient manufacturers that participated in the survey indicated the following experience prior to the relevant IVTT (or their first IVTT activity if multiple observations were provided):

- 4/10 (40%) had prior experience as a TT recipient for any vaccine;
- 1/10 (10%) had prior experience as a TT supplier for any vaccine;
- 2/10 (20%) had prior experience with producing influenza vaccines.

*Prior experience*: During interviews, several recipients stressed that although they may not have had previous experience with TT, the IVTT experience helped them be more efficient in TTs that followed. A supplier highlighted the importance of the recipient’s experience with manufacturing any other product prior to IVTT because the familiarity with production processes and quality control made the transfer more efficient, which was echoed by several recipients and experts. One recipient noted its prior influenza vaccine production know-how was the most important determinant for IVTT success and the main motivation to upgrade its previous facility. For two recipients that had no prior experience with influenza vaccine production, the relevant IVTT allowed the companies to realize that the lack of relevant human capital was a major challenge.

*Management structure*: Several manufacturers and experts commented on differences seen between recipients that were private versus those that are public, including differences in motivation (e.g., revenues versus public value), a tendency for greater technology absorption capacity among private entities, and lengthier procurement processes among public entities.

#### 3.3.2. Transfer Supplier

Two suppliers, both of which are private companies, participated in the survey. Prior to IVTT, neither had prior experience as a TT supplier for any vaccine but both had prior experience as a TT recipient.

*Supplier commitment and stability*: Recipients stressed the importance of the commitment level from the supplier and the need for a true partnership. The level of experience for the supplier was mentioned often, especially for ensuring the receipt of good quality processes and products, which helps reduce issues with approval by the recipient’s NRA. Like the recipient, stability at the supplier site was highlighted as a strength.

*Utility of expert consultants*: Regarding the transfer of know-how within the WHO TTI, experts acknowledged a key strength was the deployment of consultants with a range of expertise from generic manufacturing to specific process issues.

#### 3.3.3. Transfer Object

**Figure 2:**
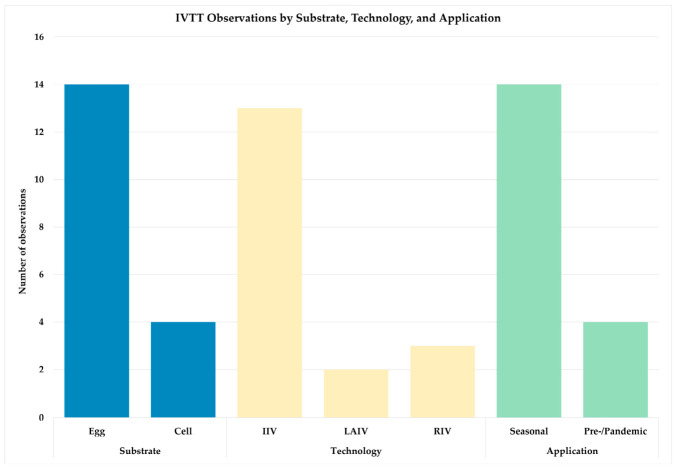
IVTT observations by substrate, technology, and application.

The overall set of IVTT observations reflects a high degree of heterogeneity in transferred technologies, ranging from long-established egg-based IIVs and LAIVs to newer cell-based IIVs and RIVs. Figure 2 provides an overview of the observations, broken down by production substrate (egg or cell), technology (IIV, LAIV, or RIV), and application (seasonal influenza or pre-/pandemic influenza). When combining substrate, technology, and application, egg-based seasonal IIVs were the most frequent observation (9/18, 50%). Three IVTT observations included an enhanced influenza vaccine, including two RIVs and one adjuvanted IIV.

*Specialized nature of influenza vaccines*: Participants discussed the specialized nature of influenza vaccines, including the sourcing of eggs, which was the reason why one recipient chose to pursue cell-based over egg-based influenza vaccine production. One expert noted that with influenza vaccines, there is high economic interest and traction but limited willingness to evaluate newer technologies. A recipient that produces an RIV made a similar observation and noted that regulatory approvals were delayed because the NRA was not familiar with the technology and did not have experience with assessing it. One expert noted that an adjuvant strategy must be developed from the start of the IVTT process given adjuvants are an important addition to some pandemic influenza vaccines.

*Seasonal versus pandemic application of technology*: One manufacturer noted that the IVTT was a good choice because there is always a need for the vaccine, especially due to the pandemic potential. One expert summarized an issue with influenza vaccines and engaging in IVTT, noting that if there is a need to protect against seasonal influenza, then it may be better to purchase it internationally if it is much less expensive and national demand is low. However, if the need for the technology is driven by pandemic preparedness, then countries and manufacturers need to question if the technology used for seasonal influenza vaccines will be the one used during a pandemic. This expert said:

Because the issue with seasonal influenza vaccine production is eggs. What can you do with eggs? Not much…If the purpose is preparedness, I would argue that technology transfer should focus on other technologies which are more versatile than what is currently the case for influenza.

#### 3.3.4. Transfer Media

**Figure 3:**
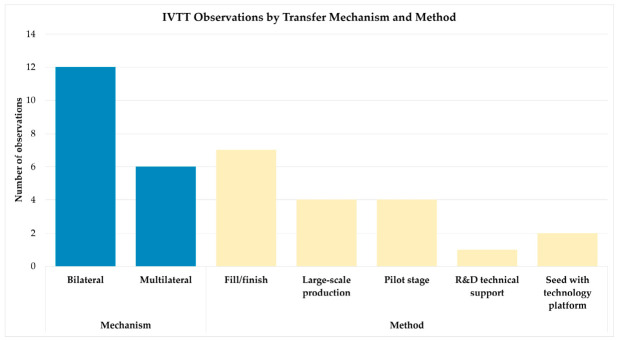
IVTT observations by transfer mechanism and method.

Figure 3 provides a breakdown of the 18 IVTT observations by the transfer mechanism and method. Bilateral agreements accounted for 12/18 observations (67%) while the WHO TTI was the mechanism for the remaining 6/18 (33%). Fill/finish was the most often used method (7/18, 39%), followed by large-scale production (4/18, 22%) and pilot stage (4/18, 22%) transfers.

*Nuts and bolts*: Participants discussed the transfer media, with one expert referring to this component as the “nuts and bolts” of the IVTT project, including understanding the vaccine production processes, equipment, and facilities. One expert noted that the mechanism is critical and can help overcome a lot of difficulties, such as lack of experience of the supplier or recipient. Several participants highlighted how important training, including hands-on training at the supplier’s site, is to the transfer media and should be conducted throughout the project.

*Clear terms and motivations for the transfer*: The issues of a strong relationship between the supplier and recipient and clear and well-defined terms of the transfer were highlighted by participants. There was a focus on the need for defined criteria of success and metrics from the start, regular check-ins throughout the project, milestones, go/no-go decisions, and feedback on how the technology is used. Participants highlighted the importance of contracts between TT partners, including instances where the supplier benefited from the inexperience of the recipient which led to a flawed agreement and little incentive for the supplier to accelerate the process, thus highlighting the importance of the recipient’s business development experience. One private manufacturer described the approach to establishing a public–private partnership for IVTT. During the 2009-10 influenza A(H1N1) pandemic, the manufacturer approached its national Ministry of Health (MoH) with its IVTT proposal to secure interest and help stimulate government support; this resulted in interest from a supplier and an agreement with clear milestones and timelines driven by MoH requirements and expectations.

*Multilateral transfer*: Experts discussed the top-down approach from WHO through the GAP and TTI that influenced uptake of the technology by some manufacturers, even though the demand was not necessarily evident. Most TTI participants did not identify a supplier, so the mechanism involved sharing of know-how through the IVTT hub at NVI or by vaccine production experts. One expert highlighted how important it was to include prior vaccine production as a key criterion for participation in the TTI. Several experts discussed the parallel TT of adjuvants that allowed for an antigen agnostic approach to capacity building with the recipients but was tied back to the IVTT activities. Two separate criticisms of TTI were highlighted. First, one expert suggested that the program should have included a decision-making process for when to withdraw funding from those not making progress and made the argument for a model of giving more to fewer manufacturers given the amount of effort and funding required. Second, one expert noted that more attention to clinical trials would have been helpful at the beginning, including defining the design, resources, and evaluation criteria.

*Value of fill/finish*: One recipient already had experience doing fill/finish with other vaccines so receiving it for influenza was not new, echoing a preference by some suppliers for recipients to start with fill/finish. Another recipient described the benefit of beginning with labeling and packaging and moving to filling and formulation, with additional profit margins experienced with each phase.

#### 3.3.5. Demand Environment

Seven of the 10 recipients (70%) indicated that there was a national seasonal influenza vaccination policy and/or influenza vaccines were used in the country prior to IVTT. One supplier indicated that the recipient country used influenza vaccines prior to the IVTT. Only 1/10 recipients (10%), which was a public manufacturer that participated in the WHO TTI, reported receiving financial support from the government for their IVTT activities, although it has been reported previously that WHO seed funding helped catalyze government financial support for many of the TTI participants [18].

**Figure 4:**
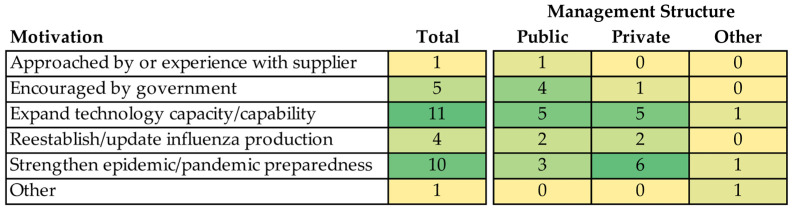
Heatmap of IVTT motivations, including by management structure of the transfer recipient or supplier.

Manufacturers indicated their motivations for pursuing IVTT. Figure 4 shows a heatmap of total IVTT motivations and motivations stratified by the management structure of the transfer recipient or supplier. Expansion of the technology capacity/capability and strengthening epidemic/pandemic preparedness were the most common motivations for manufacturers. While both public and private manufacturers noted expansion of the capacity/capability as a motivation, differences were seen where more public manufacturers indicated encouragement by the government while more private manufacturers indicated strengthen epidemic/pandemic preparedness. One manufacturer reported encouragement by WHO as another motivation.

*Market certainty and predictable demand*: All participants discussed the importance of the demand environment and its role in attracting IVTT opportunities and influencing success. One recipient noted that the only driver for the supplier was the market in the recipient country, and if the market had been larger, the supplier may have been more engaged to support the IVTT. This same recipient noted that without market certainty and demand, it will be hard to convince anyone to focus on IVTT, especially given influenza vaccines are not a priority in many countries; this was echoed by a supplier suggesting that the presence of the vaccine market in the recipient country was the only reason why the IVTT was completed. A recipient also noted that having a multi-year contract in place with the MoH with clear supply volumes and price helped maintain confidence in the project. Another recipient leveraged the market needs to push for changes at the transfer supplier such that the bulk product for fill/finish could be more appropriate for the local context (e.g., Halal). Regarding the WHO TTI, one recipient noted that there was a large focus on the push factors, such as skill development, infrastructure, and regulatory capabilities, but not enough focus on the market aspects.

*Political will for TT*: One expert noted, “if [political will] wasn’t 95–100%, then it just made it very difficult…for the company to even accept tech transfer.” One recipient provided an example where government support of previous TT projects for other vaccines through a government-to-government program paved the way for the IVTT project. A public manufacturer noted the government’s financial support for procuring equipment but lacked support for the development of the sustainability strategy.

*Seasonal vaccination versus epidemic/pandemic preparedness tensions*: Several participants noted that demand following the 2009-10 A(H1N1) pandemic was a driver for establishing the capacity, which aligns with the survey data showing strengthening epidemic/pandemic preparedness as a common motivation. Participants commented on the tensions for motivations related to epidemic/pandemic preparedness versus those related to expanding capability/capacity, especially for seasonal influenza. One manufacturer underscored how these motivations were not synchronized by describing the ideal preparedness model as one “where you say all I need to do is build capacity so that I’m ready for a pandemic and there is a magical mechanism to support me while I wait for the next pandemic” versus the reality that involved building “a routine production platform [that] needs routine vaccines and those routine vaccines can only be considered routine if there’s a market that routinely takes its vaccines up.” One expert expanded on these tensions, noting that the business model of emphasizing the pandemic threat to promote influenza vaccination was reinforced by the 2009-10 pandemic and global policies that followed, but markets no longer exist when the threat is gone, resulting in companies wasting vaccines.

### 3.4. Effectiveness Criteria

#### 3.4.1. Effectiveness Related to Commercial Benefits

Nine of the 10 recipients completed at least one IVTT agreement, and the one supplier providing additional data completed its IVTT agreement. Fifteen of the 18 IVTT observations (83%) were completed, 2/18 (11%) were terminated, and 1/18 (6%) was ongoing at the time of the survey. For each agreement, participants indicated the year it was initiated or signed with the supplier and the year it was completed or terminated. For the 15 completed agreements, the mean time to completion was 4.5 years (median: 3; range: 1–15), and for the two terminated agreements, the RIV transfer was terminated after three years and the seasonal IIV transfer after five years. The associated IP was transferred with the know-how for 5/15 observations (33%); four of these observations involved transfers of seasonal IIVs and one involved a seasonal LAIV. Notably, one of the IIV observations involving transfer of IP was reported by a supplier.

Thirteen of the 15 completed IVTT observations resulted in an approved vaccine (87%), including 10 seasonal influenza vaccines and three pre-/pandemic influenza vaccines. The mean time from IVTT initiation to approval for the 13 vaccines was 5.3 years (median: 5; range: 1–13). Three of the 10 approved seasonal influenza vaccines (30%) were exported.

To understand if there were differences in times to IVTT completion and influenza vaccine approval, the mean number of years was calculated for nine variables across the determinant domains: prior TT experience, prior influenza vaccine production experience, and management structure (transfer recipient); technology and application (transfer object); mechanism and method (transfer media); and prior influenza vaccine policy/use (demand environment). The change in mean number of years was calculated between a comparison group and reference group. Transfer supplier characteristics were not captured for each IVTT observation and are therefore not included in the calculations. Additionally, because only one manufacturer reported receiving financial support for IVTT from the government, this variable was excluded from the demand environment domain.

**Figure 5:**
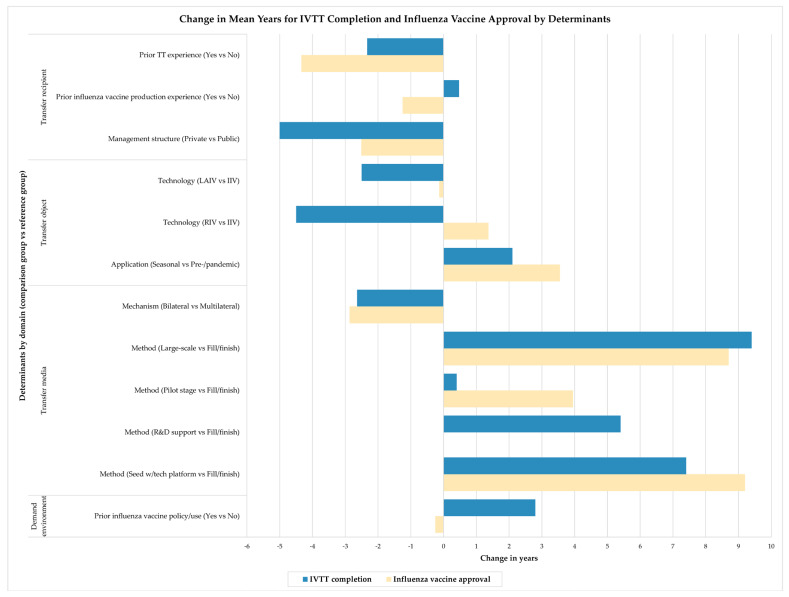
Change in mean years for IVTT completion and influenza vaccine approval by determinants. Note that the one manufacturer pursuing the R&D technical support transfer method did not report achieving vaccine approval, and therefore that data is excluded for the influenza vaccine approval timeline.

Figure 5 shows the change in mean number of years for IVTT completion and influenza vaccine approval, stratified by determinants. Key findings include:

Transfer recipient

- Manufacturers with prior TT experience and private manufacturers reported shorter timelines.
- While manufacturers with prior influenza vaccine production reported longer timelines for IVTT completion, they reported shorter timelines for influenza vaccine approval.

Transfer object

- When comparing LAIV and RIV to IIV, manufacturers reported shorter timelines for IVTT completion. Vaccine approval timelines were longer for RIV.
- Manufacturers reported longer IVTT completion and vaccine approval timelines for seasonal versus pre-/pandemic influenza vaccines.

Transfer media

- Manufacturers participating in bilateral mechanisms reported shorter timelines, although it should be noted that those participating in the WHO TTI often pursued more complex transfer methods rather than fill/finish.
- Using fill/finish as the reference, manufacturers pursuing large-scale production, pilot stage, R&D technical support, and seed with technology platform methods reported longer timelines, although the time to IVTT completion was only slightly longer for pilot stage versus fill/finish.

Demand environment

- Manufacturers that reported the existence of influenza vaccine policies or use prior to IVTT were shown to have longer IVTT completion timelines but slightly shorter vaccine approval timelines.

*Focus on commercial benefits over spillover benefits*: Participants highlighted the importance of the commercial effectiveness of IVTT. A supplier noted their company considers direct, material benefits more important than spillover benefits. A recipient noted:

The very purpose of a technology transfer is to have a commercial impact, otherwise you don’t do it…Ultimately, it’s the ability to get across the finish line, have a product on the market, and you can generate revenue, which makes you more sustainable, and you can be around tomorrow.

*Out-the-door criterion*: One expert noted that IVTT projects were typically longer than originally expected and six to seven years became the standard. A separate expert noted that the demand environment will not actually affect the transfer, nor will it compromise the quality of the IVTT. Although one recipient did not provide specific details for the RIV terminated agreement, when referencing that transfer, they described the process of reviewing and selecting the appropriate technology, emphasizing that taking the time to fully understand the technology allows for better decision-making for investments and identification of the supplier. The other recipient with a terminated agreement noted challenges related to the company’s limited experience with TT, difficulties validating the technology, and minimal engagement from the supplier, stating: “I would argue that had our market been a 60 million-dose market, [the supplier] would not have the attitude of saying, ‘Listen, try to sort it out.’”

*Impact of approved product*: One recipient noted that once their vaccine was on the market, the internationally supplied vaccine became 50% cheaper coming in just 20 cents below the local product. Another recipient noted that the MNC supplying influenza vaccines to the country stopped trivalent influenza vaccine (TIV) production completely which allowed the recipient to become the sole supplier of TIV, supplying 100% of their product to the annual influenza vaccination campaign. WHO PQ was mentioned as an important driver to support market impact, including finding partners to invest in further development of influenza and other vaccines. While several experts noted that IP rights were not an issue with the WHO TTI given it involved long-established technologies, one recipient noted how critical this issue will be for NGIVs, including for mRNA vaccines where “development is very restricted and limited due to the patent issue.”

#### 3.4.2. Effectiveness Related to Non-Commercial Benefits

**Figure 6:**
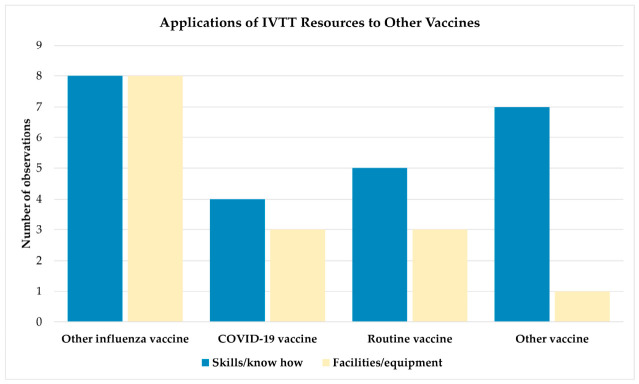
Applications of IVTT resources to other vaccines.

Participants provided partial information on the non-commercial benefits for the IVTT observations, including training, opportunity costs, political advantage, and public value. Staff at the recipient manufacturer received training for 10/12 (83%) of those agreements. Opportunity costs were reported by recipients for 11 IVTT observations by indicating if resources derived from IVTT (i.e., skills/know-how or facilities/equipment) were leveraged for the development and production of other influenza, COVID-19, routine, or other vaccines. Figure 6 shows that the skills/know-how and facilities/equipment from the majority of IVTT observations (8/11, 73%) were used for the development of other influenza vaccines. For COVID-19, routine, and other vaccines, skills/know-how were leveraged more often than facilities and equipment.

Regarding political advantage, recipients indicated that government financial support followed the successful completion for 2/11 IVTT agreements (18%), which were both completed by public manufacturers.

Regarding public value, 3/11 IVTT agreements (27%) resulted in a dedicated government procurement agreement, two of which were associated with a private manufacturer and one with a public manufacturer. Two of the 10 approved seasonal influenza vaccines (20%) were supplied through regional/global procurement mechanisms. Annual production capacities were provided for 11 completed IVTT observations. For the 10 seasonal influenza vaccines analyzed, the total annual production capacity was approximately 226.5 million doses, which accounts for 15% of the estimated global annual seasonal influenza vaccine production capacity of 1.53 billion doses [31]. The average production capacity was 22.6 million doses, which was skewed by a small number of high-capacity manufacturers, as indicated by a median of 6 million doses.

*R&D and business development benefits*: One expert noted that non-commercial benefits are almost as important as the actual delivery of a vaccine:

It’s creating so many collateral benefits outside of the object. It’s changing the world, it’s impacting people…The expertise, the collateral benefit, in terms of collaboration opportunities, common markets, is key, is number one in the end. It’s not what you look for, but this is what you gain.

Several manufacturers described how the IVTT experiences supported the development of other vaccines, including for pre-pandemic influenza A(H7) and A(H9) vaccines. Additionally, the IVTT experience provided opportunity costs in terms of business development experience. However, one expert did not think the non-commercial benefits were realized fully because influenza is not a priority for some manufacturers and countries.

*S&T human capital*: There was general agreement among participants of the positive impact on human capital. One recipient highlighted that human capital development cannot be overstated because with each TT, human capital increases exponentially. However, as one expert explained, this comes at a cost as highly trained staff will get recruited by competitors, underscoring the importance of staff retention. One expert highlighted the reciprocal, synergistic benefit for both the recipient and supplier in terms of human capital, especially if the two partners do not have the same management structure or company size. This same expert emphasized the importance of sharing information back to the supplier (e.g., what worked, was the technology used, did it go to clinical trials), which also enables the supplier to grow its experience. The human capital benefits beyond the recipient was also highlighted for regulatory staff, with a recipient noting that preclinical trials for their quadrivalent influenza vaccine were done much more quickly because of the IVTT experience with their TIV.

*Political advantage*: Political advantage was recognized as an important driver for future TT because it can help the recipient attract attention from potential partners. One public manufacturer noted that the IVTT success favorably positioned the company within the national health system. Similarly, a private manufacturer highlighted greater recognition by the MoH and NRA and that the MoH has used the IVTT success to publicize the benefits of transferring technology in the country. A private manufacturer detailed government financial support for clinical trials for the vaccine resulting from the IVTT as well as financial support for other vaccines using the same platform technology. A public manufacturer noted the political advantage was important because the country has a national development program whereby the government prioritizes the nationally produced vaccine even if it is a higher price. Regarding the countries with manufacturers that participated in the WHO TTI, experts highlighted variable political advantage due to changing of governments and inconsistent commitment throughout the projects.

*Public value*: The public value of the IVTT successes was noted by several participants, with one describing the value of bringing a high quality, low cost, locally produced vaccine to the public relatively quickly. Public awareness was highlighted by one recipient as a critical component of IVTT effectiveness and was an obstacle due to public perception that a locally produced vaccine is of lesser quality, which could be mitigated by WHO PQ to garner public trust and acceptance. One recipient described how the first IVTT experience helped drive improvements in national vaccination logistics and vaccination center trainings, and this value was noticed during the COVID-19 pandemic since many of the systems were already in place. Regarding the WHO TTI, one expert noted that the desired result in most cases was not the product itself but rather the capacity, which was special for influenza and not necessarily the desired result for most other TT projects. This sentiment was further emphasized by one expert noting that although one of the larger TTI participants discontinued production of its influenza vaccine, the manufacturer still has the technical capacity and could scale up relatively easily, especially based on previous marketing authorization experience.

### 3.5. TT Enablers

Participants assessed the level of importance of each of the 17 TT enablers using a Likert scale, with 1 being “not at all important” and 5 being “critically important”. One Likert response for the enabler “Proper access to information” was missing from a recipient manufacturer; the value was imputed using that sub-group’s median. Another response marked as not applicable for “IP-friendly environment” was retained as missing and excluded from relevant pairwise analyses. Table 4 provides descriptive statistics for all 17 enablers. Median scores range from 4 to 5, suggesting all 17 enablers are considered important to a certain degree as they were above 3, which was designated as “neither important nor unimportant” in the Likert scale. Three enablers (adherence to regulatory standards; resource prioritization at the company level; and workforce recruitment, training, and retention) were marked as important by all participants as indicated by minimum values of 4. By contrast, “access to appropriate capital markets” and “adherence to ethical standards” showed variation in responses as these two enablers had the largest ranges. Document S8 in the Supplementary Materials provides a detailed summary of the enablers, including mean Likert scores, standard deviations, and interquartile ranges.

Mann–Whitney U tests resulted in no statistically significant differences in perceived importance of the 17 enablers between experts and manufacturers. “Adherence to ethical standards” had a marginally non-significant difference (*p* = 0.056), with mean ranks showing that manufacturers tended to rank it as more important than experts. Kruskal–Wallis H tests resulted in a statistically significant difference among participants only for “adherence to ethical standards” (*p* = 0.010). Post hoc pairwise analyses showed a significant difference in perceived importance for experts versus recipients (*p* = 0.014, Bonferroni-adjusted), with recipients rating this enabler more highly.

In addition to assessing the level of importance of the 17 enablers individually, participants were asked to rank the top five enablers overall. Rankings were weighted and sums across all participants were calculated (see Table 4). All enablers were ranked in the top five by at least one participant, as indicated by a sum greater than zero. The following five enablers had the highest weighted sums: government commitment to local production and procurement, adherence to regulatory standards, viable and accessible local market, adherence to ethical standards, and functional NRA. Document S9 in the Supplementary Materials provides a detailed summary of the weighted ranks of the enablers, including mean rankings, standard deviations, and interquartile ranges.

Mann–Whitney U tests resulted in a significant result for “workforce recruitment, training, and retention” (*p* = 0.047), with experts ranking this higher than manufacturers. Kruskal–Wallis H tests resulted in a statistically significant difference (*p* = 0.020) in the weighted ranking of “government commitment to local production and procurement of vaccines” among participants. Post hoc pairwise analyses showed that the difference was driven by a contrast between experts and suppliers (*p* = 0.017, Bonferroni-adjusted), with experts assigning higher average rankings.

Interviewed participants tended to place more emphasis on the following enablers: government commitment to local production and procurement; viable and accessible local market; common values, trust, and commitment from all parties; functional NRA; and adherence to regulatory standards.

*Regulatory environment*: One participant noted that adherence to regulatory standards must be in place for the partnership to begin, with another participant noting that it is not necessarily an enabler but rather an expectation. One recipient emphasized the importance of regulatory standards because they are regularly questioned if their product is the same quality as those supplied by MNCs. Several participants noted the importance of the relationship between the NRA and recipient and that the NRA should be engaged from the beginning, especially if it is a new product or technology. This relationship was highlighted as particularly salient for influenza given the need for the NRA to release vaccine batches every year in a timely manner. One recipient noted regulatory delays because the NRA did not have the necessary preclinical and clinical trial expertise.

*Sustained government commitment*: All interviewed participants agreed on the importance of this enabler, including for creating and expanding the local vaccine market. One public manufacturer noted struggles with maintaining government commitment, which has resulted in the manufacturer not being recognized as a national vaccine producer and thus public health institutes and healthcare professionals are not familiar with the manufacturer’s influenza vaccine. An expert noted that one manufacturer failed solely because the government was not behind it. Some participants noted the specific relevance of government commitment to influenza vaccines for two reasons: (1) annual influenza vaccine production and procurement timelines do not allow for a lot of time to negotiate contracts, so government understanding of influenza and its seasonality is key; and (2) influenza vaccines are not a standard product in vaccination programs in most countries, thus they are not supported by global procurement mechanisms (e.g., Gavi). Another expert noted the shift in government commitment to local production from a focus on economic or public health effectiveness to security, where governments may justify larger premiums to maintain vaccine facilities even if they are not in use. One recipient noted it was important to focus on regional markets. After the 2009-10 A(H1N1) pandemic, there was a push from a lot of countries to have local production, which was not feasible for every country, so this manufacturer explored opportunities to expand its market in multiple countries in the region.

*IVTT partnerships*: Participants commented on the importance of common values, trust, and commitment from all parties not only for the relationship between the recipients and suppliers but also for the success of the IVTT, which would include the government and NRA. One recipient noted that the relationship with the supplier was important because they also supply the antigen for fill/finish production; having a good relationship with and commitment from the supplier helps to mitigate risks and allows the recipient to leverage the supplier’s network if needed.

### 3.6. Additional IVTT Recipients and Suppliers

Additional IVTT observations for eight recipients and one supplier were identified through the literature (Table 5). Document S10 in the Supplementary Materials provides a detailed overview of the determinants and effectiveness criteria for the observations.

Six of the eight recipients (75%) were public institutions and 2/8 (25%) were private. Most had prior experience in the production of non-influenza vaccines, with the exception of the Mechnikov Institute in Nicaragua, which was established to produce influenza vaccines for the country and region. The predominant transfer object was egg-based IIV; others included egg-based LAIV, cell-based IIV, and a complementary transfer of an oil-in-water adjuvant. Transfer media varied with two recipients engaging in bilateral agreements only, four participating exclusively in the WHO TTI, and two utilizing hybrid approaches where the WHO TTI participation complemented the bilateral IVTT agreement. The transfer methods included fill/finish, pilot-scale, stepwise, and full-scale production capabilities. Available information for the demand environments varied. However, highlights included only one instance of an NRA being considered functional prior to the IVTT, three countries were using influenza vaccines prior to the IVTT, and one country’s pandemic preparedness plan specifically called for the establishment of domestic seasonal influenza vaccine production.

For effectiveness outcomes, 5/8 IVTT observations (63%) were completed, 1/8 (13%) had an unknown status, and 2/8 (25%) were terminated, although partial progress in the form of laboratory-scale production of A(H1N1) vaccines was reported in one of the terminated observations. Four of the eight recipients (50%) (two public and two private manufacturers) achieved approval of at least one influenza vaccine, with the Serum Institute of India (SII) being the only manufacturer to achieve multiple approvals across technologies and seasonal and pandemic applications. Two private manufacturers (Mechnikov Institute and SII) also achieved WHO PQ for at least one product. Reported timelines from IVTT initiation to vaccine approval showed shorter periods for pandemic vaccines (1–2 years) compared to seasonal vaccines (5–6 years), with WHO PQ pursuit adding an additional 2 years for SII’s pandemic vaccine and 1 year and 6 years for SII’s and the Mechnikov Institute’s seasonal vaccines, respectively. Human capital development was reported for 7/8 (88%) recipients. Additional outcomes included opportunity costs, such as pursuit of other influenza vaccines and COVID-19 vaccines, political advantages in the form of government funding for clinical trials, and broader public value, including reported pandemic production capacities exceeding 260 million doses of monovalent influenza vaccines across two manufacturers.

NVI, which served as a central hub for the WHO TTI, represented a key supplier in the IVTT landscape for both the institutes identified in the literature search and those that participated in the survey and interviews. As a public institution with prior experience in vaccine production and TT, NVI supported multiple recipients through generic and targeted trainings. In addition to direct product-related outcomes, NVI contributed to human capital development across many institutes and even participants from NRAs.

A diverse set of enablers and barriers was identified with several common themes. Access to capital was a critical enabler across multiple observations, often facilitated through grants provided under the WHO TTI coupled with national investments. Regulatory capacity-building efforts led to the establishment of functional NRAs in several countries, including Egypt and the Islamic Republic of Iran, despite the IVTT terminations by the recipients in those countries. The lack of IP barriers associated with whole and split virion egg-based IIV production facilitated IVTT through the NVI hub, but IP barriers, particularly related to reverse genetics in LAIV development, were cited. Other technology- and production-related barriers included access to necessary equipment and the sourcing of eggs and vaccine viruses. Vaccine market limitations were also noted, particularly in the case of SII where the absence of seasonal influenza vaccination policies and weak demand were barriers to sustained production and market impact despite previous government support for pandemic vaccine production and supply.

## 4. Discussion

In this study, we characterized the effectiveness of past IVTT activities using a contingent effectiveness model for TT, assessed the relationship between determinants and effectiveness criteria, and identified enablers considered important for successful TT. Primary data were collected from participants mostly representing IVTT recipient manufacturers and experts, with just two suppliers providing data on IVTT experiences and enablers and another supplier only providing data on IVTT enablers. However, five experts reported providing direct technical assistance to recipients and offered insights that may partially reflect the supplier perspective. The experts represented a broad range of technical and institutional perspectives. In addition to diverse areas of expertise spanning manufacturing, product strategy, public health, regulatory issues, R&D, and TT, their professional backgrounds encompassed multiple sectors, including academia, funding agencies, international organizations, private sector, public sector, NRAs, and the IVTT and adjuvant TT hubs supporting the WHO TTI. Secondary data were collected for an additional nine institutions, representing eight recipients and one supplier. Together, the inclusion of secondary data enabled triangulation of findings across independently reported IVTT experiences and provided additional context for successes and challenges.

As this mixed-methods study was exploratory, the small sample provided insights in the effectiveness of IVTT rather than inferential conclusions. The contingent effectiveness model allowed for a structured review of past IVTT observations. Vaccine manufacturers provided primary data on 18 unique IVTT observations. The observations reflected substantial heterogeneity across technologies, which may help explain differences observed in the nature of IP transfer versus know-how exchange, variation in regulatory pathways including familiarity with newer platforms, and timelines for vaccine approval. Commercial effectiveness was demonstrated through the completion of 15/18 IVTT agreements that resulted in 13 approved vaccines at the time of the study. Non-commercial effectiveness was demonstrated through training of staff for the majority of IVTT observations and the application of IVTT-derived skills/know-how and facilities/equipment to the development of other vaccines. Political advantage was not demonstrated widely as only two IVTT observations resulted in government financial support and only three observations resulted in procurement agreements. However, the secondary data obtained through the literature suggested that political and public value outcomes may be more pronounced in certain contexts, including government financial support for clinical trials and pandemic influenza vaccine production rather than seasonal influenza vaccine production.

Experts and manufacturers largely agreed on the absolute and relative importance of the 17 enablers, with an emphasis on the mechanics of the transfer (e.g., common trust and commitment among all parties, workforce), regulatory approvals (e.g., functional NRA, adherence to regulatory standards), and sustainability (e.g., government commitment to local production, viable and accessible vaccine market). The agreement was supported by non-parametric tests, which only resulted in statistically significant differences for the adherence to ethical standards; workforce recruitment, training, and retention; and government commitment to local production and procurement of vaccines enablers. Experts ranked these enablers higher than manufacturers, and while these results should be interpreted cautiously given the small sample size, they may reflect differences in how the participating experts experienced IVTT as implementers, suppliers, or technical advisors. These findings highlight the complexities of TT among those manufacturers participating in the study and suggest that effectiveness may not be limited to whether it resulted in an approved vaccine. These patterns were further supported by the secondary data, which similarly emphasized higher rates of IVTT completion but few vaccine approvals and consistent evidence of human capital development across diverse institutional settings.

Differences in timelines for IVTT completion and influenza vaccine approval were observed across recipient, object, media, and demand domains. Manufacturers with prior TT experience and private management structures tended to complete transfers more quickly, while prior influenza vaccine production experience was associated with shorter approval periods. Shorter timelines were associated with LAIV and RIV technologies and bilateral mechanisms. Although timelines could only be calculated for two institutes represented in the secondary data, similar patterns were seen for pandemic vaccine versus seasonal vaccine approval timelines. While IVTT completion may reflect the supplier, recipient, and media dynamics, vaccine approval timelines may capture broader factors, such as national policy recommendations for influenza vaccination and maturity of regulatory approval pathways that influence the pace at which transferred technologies translate into licensed products.

Two IVTT agreements reported in the survey were terminated prior to completion, with meaningful lessons that influenced future TT agreements. One recipient suggested that the IVTT termination was driven by strategic considerations related to the suitability of the technology itself, highlighting the importance of aligning technology selection with institutional capabilities and long-term objectives. The other recipient cited multiple factors, including minimal engagement from the supplier, which contrasted with later, more successful TTs with suppliers that would deploy technical teams to troubleshoot issues onsite. While the available literature for the two terminated agreements in the secondary data did not indicate specific reasons for the termination, a combination of reported technical, financial, and institutional constraints may have influenced the termination [18]. These findings suggest that multiple determinants may interact to shape positive and negative IVTT outcomes, demonstrating the model’s value for exploring such interdependencies. Given there is no one-size-fits-all approach to TT, this recognition of the interconnectedness among determinants may be an important component of future TT agreements, such that targeted technical and policy support may help overcome challenges for certain determinants. Regarding this interplay of determinants, one expert said:

I think that all five determinants are important, that they don’t all have to be at maximum value if you wish to start…Weakness in one of the determinants can be, to a certain degree, compensated by strength in the other one, but you need to have some positive aspects in all of them.

Although one expert noted that the demand environment may not impact the quality of the transfer itself, insights from the survey and interviews suggest that the demand environment has a role throughout TT as a prerequisite (i.e., the motivation for pursuing TT), enabler (i.e., trust and commitment from all parties, including policymakers and regulators), and goal (i.e., the local vaccine market, R&D incentives and regulatory support for vaccine approvals). This interpretation highlights that components of the contingent effectiveness model can overlap and emphasizes the importance of stakeholder engagement throughout TT. The secondary data underscore this dynamic, with successful IVTT activities being associated with an established demand environment and government commitment, including pandemic preparedness plans, procurement agreements, and policy support for domestic production. The role and impact of the government should not be ignored, with one expert noting:

Tech transfer is always hard, no matter what, in any environment. Even within one company, a tech transfer from one facility to another is very challenging…What seemed to make the biggest difference in terms of who was able to succeed and who had challenges, the secret sauce seemed to be the relationship of the recipient with the government.

Limited or uncertain demand environments have been associated with significant impacts on IVTT outcomes, including in the case of SII where, despite successes in vaccine approvals and WHO PQ compared to other IVTT recipients, the lack of influenza vaccination policies and limited demand challenged sustainability of the manufacturer’s seasonal influenza vaccine program. This finding could inform future discussions on TT so that conversations go beyond the transfer itself and focus on the role of each stakeholder in ensuring the effectiveness of TT. Given the focus on TT in pandemic preparedness, these findings may support additional analyses and discussions related to ensuring robust demand environments, sustaining stakeholder commitment, and adapting flexible transfer mechanisms that can operate under emergency conditions. Additionally, these findings position the demand environment as a component that may shape the trajectory and sustainability of IVTT outcomes.

The WHO TTI and its impact have been described before [18,19,64], but this study allowed for further analysis of the program, especially in the context of bilateral agreements, because 8/14 TTI manufacturers provided primary data for the study and secondary data from the remaining 6/14 manufacturers were included. Additional secondary data from the literature, including analyses of the NVI as a central hub, further highlight the role of coordinated training and technical assistance in enabling downstream vaccine approvals and human capacity development across multiple recipients. Although IVTT completion and vaccine approval timelines appeared longer for the TTI participants, this may be driven by the selection of technologies and the use of more complex transfer methods beyond fill/finish. As of 2023, TTI manufacturers were able to contribute 14% of the annual global seasonal influenza vaccine production and 7% of the potential global pandemic influenza vaccine production [31], and as of March 2026, three TTI manufacturers had signed PIP Framework benefit-sharing agreements for the real-time supply of 10% of vaccines in the event of an influenza pandemic. These data highlight the effectiveness of the TTI beyond just the number of vaccines approved throughout the program, especially for the global public value added by this initiative and contribution to human capital development and regulatory strengthening observed across primary and secondary datasets. As one expert noted, “[TTI] was actually successful, it actually did what it said it was going to do, which you can’t always say for a program.” The successes and challenges of TTI have informed the development and implementation of the mRNA Technology Transfer Programme [65], which includes some of the same manufacturers that participated in TTI, allowing for their IVTT experience to influence their mRNA TT activities. These findings suggest that lessons from IVTT may be relevant to other vaccine technologies, including next-generation platforms, when TT is paired with enabling conditions that support sustainable local production in LMICs, including government commitment, regulatory capacity, skilled human capital, viable demand, and alignment with national or regional public health needs.

### 4.1. Strengths and Limitations

Although the study achieved modest survey response rates for manufacturers and experts (43% and 48%, respectively), a limitation of this study was the small sample size. IVTT observations were skewed more towards those with overall successful outcomes and weighted more toward recipient rather than supplier perspectives. Additionally, only one MNC participated in the study. The purposeful sampling strategy was appropriate for identifying institutions and experts with direct IVTT experience, particularly given the limited documentation of such activities. However, it may have introduced selection bias because participants were identified through published sources, institutional networks, and expert knowledge of IVTT activities. Nonresponse bias was also possible as manufacturers and experts that chose not to participate likely differed from participants in their IVTT experiences and motivations, institutional capacities, or willingness to discuss challenges. Because IVTT can involve commercially sensitive aspects, regulatory challenges, and reputational considerations, response bias may have also influenced the level of detail provided by participants or contributed to more favorable accounts of completed transfers. These sampling and response limitations, together with limited representation of MNCs and suppliers, restrict the generalizability of the findings.

Despite this limitation, the sample of participating manufacturers was diverse, encompassing five WHO regions, different management structures, and a range of production capacities and capabilities as demonstrated by their current product landscapes. Seven manufacturers, including three private manufacturers, were interviewed, often discussing sensitive topics and detailed accounts of their IVTT experiences, including two terminated transfers. The willingness of manufacturers to provide comprehensive data and discuss successes and challenges reflects a high degree of transparency that strengthens the credibility and validity of the dataset. Although the expert sample was small, participants contributed perspectives from a wide range of technical and institutional backgrounds, helping to contextualize manufacturer experiences. The number of participants is comparable with other studies that incorporate case study findings or focus on one program/technology. While the potential for survivorship bias may result in more optimistic interpretations, participants identified a range of operational and policy barriers and challenges, including from both successful and terminated transfers.

To further examine IVTT, the primary data collected through the survey and interviews were complemented by secondary data compiled from the peer-reviewed and grey literature using the conceptual framework. This resulted in data on IVTT determinants, effectiveness criteria, enablers, and barriers for nine additional institutions. secondary data expanded the range of IVTT contexts captured, including both completed and terminated transfers, and provided additional representation of bilateral and multilateral transfer mechanisms. Unfortunately, the perspectives from suppliers representing MNCs were not available in the literature. The inclusion of literature-based observations does introduce its own limitations, including the variability in the depth and quality of publicly available data and potential selection and/or confirmation bias toward more documented, higher-profile IVTT activities, particularly where documented cases emphasized themes identified in the survey and interviews. To mitigate the risk, sources were screened using predefined criteria, both successful and terminated IVTT observations were included where sufficient information was available, and secondary data were compiled using the same codebook established for the interviews. The use of literature as a legitimate data source in mixed-methods studies, including its integration with quantitative and qualitative data, has been proposed as an option for further advancing mixed-methods research [66].

Another limitation relates to the retrospective design of the study that involved manufacturers and experts providing information and perspectives on past IVTT observations, including some that were initiated over 20 years ago, potentially leading to recall issues. The inclusion of secondary data from various reports and publications helped to partially mitigate this limitation as they provided independently documented accounts of IVTT activities and outcomes, including for certain institutes commented on by experts in their interviews. However, the use of the mixed-methods convergent design and inclusion of parallel concepts from the contingent effectiveness model allowed for the comparability of questions, triangulation of data, and integration of results. Including the same participants, or at least a subset, in the quantitative and qualitative research arms enabled direct comparison [67], and smaller participant samples for the qualitative research arm are not considered a problem if results are compared by topic or synthesized into a complementary picture as was done in this study [32]. The mixed-methods convergent design was particularly useful to evaluate the complexities of IVTT as it allowed for the comparison of measurable outcomes with the lived experiences of vaccine manufacturers and experts.

A final limitation was that the majority of interviews were coded by a single analyst. However, using the first version of a structured codebook, two coders assessed a subset of interviews to validate coder 1′s use of the codebook and interpretation of the data. Inter-rater reliability was assessed and all issues were reconciled through either the absorption of codes into parent codes; addition, deletion, or merging of codes; or the clarification of the definition and inclusion/exclusion criteria for codes [39]. The use of the revised, structured codebook ensured alignment with the conceptual framework, and coding focused on capturing common opinions and diverging viewpoints to illustrate experiences of manufacturers and experts, which is considered acceptable within pragmatic mixed-methods designs that value the complementarity and utility of qualitative data [32]. Data triangulation was achieved within the study by comparing survey data and interview findings and also externally through the integration of peer-reviewed and grey literature that validated and contextualized patterns observed in the primary dataset. While the level of detail available in the literature varied across institutions, the consistency of patterns across data sources strengthened confidence in the findings.

### 4.2. Future Research

The primary and secondary data provided some insights for broader TT activities. Noting the recent experiences with COVID-19 vaccine supply inequities, these findings could inform future efforts, especially for African countries that may rely on TT due to the lack of prior vaccine production capacity. While some of the findings may not be novel, the contingent effectiveness model allowed for a detailed assessment of past IVTT activities and may be considered a viable framework for future longitudinal TT research and program evaluations, or even to inform TT planning efforts. Longitudinal research designs using the contingent effectiveness model also may allow for a stronger analysis of effectiveness in terms of short- and long-term sustainability, which was not fully evident with our cross-sectional design. Additional research is needed to better understand supplier perspectives and motivations, particularly for MNCs, and how these may impact internal planning and differences in pursuit of TT across companies and even the regions they target. For this study, IP transfer was documented in only 5/15 completed IVTT observations, which may correlate with the larger number of transfers involving established influenza vaccine production technologies where know-how and process transfer predominated. However, IP rights were highlighted in interviews and the secondary data as a key consideration for the development of and access to NGIVs. Noting the emphasis of the role of IP and access to vaccine technologies in global forums, future studies of TT effectiveness should examine this more closely. Finally, given the emphasis on the government commitment and regulatory components of IVTT in the survey and interviews, future research efforts to assess TT effectiveness should also include policymakers and regulators, particularly those from LMICs, to allow for more diverse perspectives and more complete accounts of successes, challenges, and opportunities.

## 5. Conclusions

As suggested by this assessment, IVTT can be multi-faceted, time- and resource-intensive, and involve many stakeholders. While each IVTT observation was unique across the determinant domains and effectiveness criteria, several commonalities emerged, for both influenza vaccine production and broader TT processes. Several manufacturers described how their IVTT experience, in some cases representing the company’s first TT experience, strengthened their institutional capacity and enabled them to leverage the acquired expertise, infrastructure, and regulatory familiarity to pursue other non-influenza vaccines. The results align with other studies across fields that have emphasized government commitment, R&D and regulatory capacities, skilled human capital, and sustained supplier–recipient partnerships as core TT enablers. Effective TT is not universal; it will differ by recipient manufacturer, country, and even region. However, it is clear that effectiveness is contingent upon a diverse set of interconnected determinants and stakeholders, including technology suppliers and recipients, regulators, and policymakers. Effectiveness can be measured beyond the observable market outcomes of approved vaccines, including public value benefits that have societal impacts and are important for epidemic and pandemic preparedness.

TT has been identified as a global priority for improving equitable access to vaccines through the establishment of local production, especially in LMICs. This is particularly relevant in the context of ongoing negotiations of the Pathogen Access and Benefit-Sharing annex to the Pandemic Agreement, which proposes TT as a benefit-sharing option in exchange for accessing relevant biological materials and information, similar to what is included in the PIP Framework. Our findings suggest that TT effectiveness will depend not only on its inclusion as a formal commitment but also on the extent to which enabling conditions (e.g., government commitment, regulatory capacity, and viable market demand) are in place to support successful and sustainable implementation. Future TT efforts, particularly for next-generation vaccine platforms, can build on these insights to better align technological, institutional, and market conditions to achieve timely and sustainable outcomes that contribute to national, regional, and global epidemic and pandemic preparedness and equitable access to vaccines.

## Figures and Tables

**Table 1:** Contingent effectiveness model components and application to IVTT for the (a) determinants and (b) effectiveness criteria.

**(a) Determinants**		
**Domain**	**Focus**	**Application to IVTT**
Transfer recipient	Institution receiving the technology	IVTT recipient characteristics, such as prior experience with TT (as a recipient or supplier), prior experience as an influenza vaccine producer, and company management structure
Transfer supplier	Institution transferring the technology	IVTT supplier characteristics including prior experience with TT (as a recipient or supplier), prior experience as an influenza vaccine producer, and company management structure
Transfer object	Technology (content, form) that is being transferred	Influenza vaccine production technology that is transferred, including application (i.e., seasonal or pre-/pandemic) and technology platform (e.g., IIV, LAIV)
Transfer media	Mechanism by which the technology is transferred	Mechanism (i.e., bilateral or multilateral) and method (e.g., fill/finish) by which the influenza vaccine production technology is being transferred
Demand environment	Market and non-market forces connected to the need for the transferred object	Demand factors for influenza vaccines, including national influenza vaccination policy or vaccine use prior to IVTT, recipient’s motivation for IVTT, and government support for IVTT
**(b) Effectiveness criteria**		
**Criterion**	**Focus**	**Application to IVTT**
Out-the-door	An institution successfully received the technology	Transfer supplier and recipient concluded IVTT agreement (vaccine approval is not considered)
Market impact and economic development	Commercial impact of the transfer; national and regional impact beyond recipient	Combined criteria focusing on commercial impact of the IVTT, including approval of vaccine, transfer of intellectual property (IP), and export of the vaccine
Political advantage	Political reward following successful TT	Political rewards in the form of direct and indirect support to recipient manufacturer following IVTT completion
Human capital	Increase in S&T capacities and skills	Staff trained as part of the IVTT agreement
Opportunity costs	Impact of TT on alternative uses of resources or other missions of the transfer supplier or recipient	Use of IVTT outcomes and resources (i.e., know-how, facilities, equipment) for the development of other vaccines
Public value	Enhancement of collectively good and broad, societally shared values	Epidemic and pandemic preparedness, including procurement agreements, production capacities

**Table 2:** Characteristics of participating manufacturers.

Characteristics	*n* (%) or Median (Range)
Country classification by income level *	
Low-income	0
Lower-middle	3 (25%)
Upper-middle	8 (67%)
High	1 (8%)
**WHO region**	
Africa	1 (8%)
Americas	2 (17%)
Eastern Mediterranean	0
Europe	1 (8%)
South-East Asia	4 (33%)
Western Pacific	4 (33%)
**Management structure**	
Private	6 (50%)
Public (state/government owned)	5 (42%)
Public–private partnership	1 (8%)
**Annual production capacity for all vaccines (total doses)**	
<5 million	3 (25%)
5–25 million	3 (25%)
26–100 million	3 (25%)
>100 million	3 (25%)
**Product landscape**	
Seasonal influenza vaccines approved by NRA	1 (0–6)
Seasonal influenza vaccines with WHO PQ	0 (0–4)
Pre-/pandemic influenza vaccines approved by NRA	0 (0–4)
Pre-/pandemic influenza vaccines with WHO PQ	0 (0–1)
Non-influenza vaccines approved by NRA	6 (1–90)
Non-influenza vaccines with WHO PQ	0 (0–15)
**Current seasonal influenza vaccine production or product in phase 3 clinical trials**	
Yes	11 (92%)
No	1 (8%)
**IVTT role**	
Recipient	10 (83%)
Supplier	2 (17%)
**WHO TTI participant**	
Yes	8 (67%)
No	4 (33%)

* Based on World Bank classification of country income status (as of 2023).

**Table 3:** Characteristics of participating experts.

Characteristics	*n* (%)
**Primary area of expertise**	
Manufacturing	2 (18%)
Regulatory issues	1 (9%)
R&D	5 (46%)
Other *	3 (27%)
**Direct technical assistance provided to manufacturer for IVTT**	
Yes	5 (45%)
No	6 (55%)
**WHO TAG member**	
Yes	10 (91%)
No	1 (9%)

* Other areas of expertise included product strategy, public health, and TT.

**Table 4:** TT enablers: Descriptive statistics for Likert scores (1–5) of importance and weighted sums of rankings.

Enabler (Alphabetical Order)	Level of Importance			Rankings
	Median	Minimum	Maximum	Weighted Sums
Access to appropriate capital markets (i.e., project funding)	4	1	5	28
Adherence to ethical standards	5	1	5	29
Adherence to regulatory standards	5	4	5	50
Business development experience	4	2	5	9
Clear economic development priorities	4	3	5	4
Common values, trust, and commitment from all parties	5	3	5	26
Experience with evaluating the quality of technologies	4.5	3	5	11
Functional NRA	5	3	5	29
Government commitment to local production/procurement of vaccine	5	3	5	61
Innovation-friendly environment with sound IP rights	4	2	5	3
Political stability and transparent governance	4	2	5	7
Proper access to information (e.g., available resources, market opportunities)	4	3	5	1
R&D capacity of the technology recipient	4	2	5	12
Resource prioritization at the company level	4	4	5	15
Rule of Law/legal system is established and enforced	4	3	5	5
Viable and accessible local market	5	2	5	46
Workforce recruitment, training, retention	5	4	5	24

**Table 5:** Additional IVTT recipients and supplier.

Institution	Country	IVTT Role	Transfer Object	References
Birmex	Mexico	Recipient	Egg-based IIV	[19,23,41,42,43,44]
Cantacuzino Institute	Romania	Recipient	Egg-based IIV (seasonal), oil-in-water adjuvant	[18,22,23,44,45,46,47]
Mechnikov Institute	Nicaragua	Recipient	Egg-based IIV (seasonal)	[48,49,50,51]
Netherlands Vaccine Institute	Netherlands	Supplier	Egg-based IIV (seasonal and pre-/pandemic)	[20,45,52,53,54]
Razi Institute	Islamic Republic of Iran	Recipient	Egg-based IIV	[19,41,44,45]
Research Institute for Biological Safety and Problems	Kazakhstan	Recipient	Egg-based IIV (seasonal)	[23,44,45,55,56,57,58]
Serum Institute of India	India	Recipient	Egg-based IIV and LAIV (seasonal and pre-/pandemic)	[18,19,23,36,45,55,59,60,61,62]
VABIOTCH	Viet Nam	Recipient	Cell-based IIV	[23,35,36,44]
Vacsera	Egypt	Recipient	Egg-based IIV	[19,22,23,45,52,63]

## Data Availability

Anonymized data collected for this study, including survey data and interview transcripts, will be made available by request.

## Supplementary Materials

The following supporting information can be downloaded at https://www.mdpi.com/article/10.3390/vaccines14060522/s1, Document S1. Local/national manufacturers survey, Document S2. Multinational manufacturers survey, Document S3. Experts survey, Document S4. Manufacturers interview guide, Document S5. Experts interview guide, Document S6. Revised codebook for interview analysis, Document S7. Summary table of IVTT observations, Document S8. Summary table of importance rating for TT enablers, Document S9. Summary table of weighted ranks for TT enablers, Document S10. Additional IVTT recipients and supplier, Document S11. References for supplementary materials.

## Abbreviations

The following abbreviations are used in this manuscript:

DCVM	Developing country vaccine manufacturer
GAP	Global Action Plan for Influenza Vaccines
HIC	High-income country
IIV	Inactivated influenza vaccine
IP	Intellectual property
IVTT	Influenza vaccine technology transfer
LAIV	Live attenuated influenza vaccine
LMICs	Low- and middle-income countries
MNC	Multinational company
MoH	Ministry of Health
NGIV	Next-generation influenza vaccine
NRA	National regulatory authority
NVI	Netherlands Vaccine Institute
PAHO	Pan American Health Organization
PIP	Pandemic Influenza Preparedness
PQ	Prequalification
R&D	Research and development
RIV	Recombinant influenza vaccine
S&T	Science and technology
SII	Serum Institute of India
TAG	Technical Advisory Group
TIV	Trivalent influenza vaccine
TT	Technology transfer
TTI	Technology Transfer Initiative
WHA	World Health Assembly
WHO	World Health Organization

## References

[1] World Trade Organization, World Health Organization, World Intellectual Property Organization (2020). Promoting Access to Medical Technologies and Innovation—Second Edition: Intersections Between Public Health, Intellectual Property and Trade.

[2] Jadhav S., Gautam M., Gairola S. (2014). Role of vaccine manufacturers in developing countries towards global healthcare by providing quality vaccines at affordable prices. Clin. Microbiol. Infect..

[3] World Health Organization (2011). Increasing Access to Vaccines Through Technology Transfer and Local Production.

[4] Looby M., Brady C., Bentley M., Lewis A.M., Layden C., Barnoon B., Abbott E., Hughes B., Gadamasetti K., Kolodziej S.A. (2024). Three Decades of Advancements in Technical Transfer of Biologics: A Blueprint for Advanced Therapeutics. Bioprocessing, Bioengineering and Process Chemistry in the Biopharmaceutical Industry: Using Chemistry and Bioengineering to Improve the Performance of Biologics.

[5] Santos A.L.R., Wauben L.S.G.L., Goossens R., Brezet H. (2016). Systemic barriers and enablers in humanitarian technology transfer. J. Humanit. Logist. Supply Chain. Manag..

[6] Venturini K., Verbano C. (2014). A systematic review of the Space technology transfer literature: Research synthesis and emerging gaps. Space Policy.

[7] Campos N., Cortés Á.L.D.M., Pippo A.T., Rius J., Fitzgerald J., Couve A. (2025). Multiple factors shape technology transfer for the development and manufacture of vaccines in Latin America and the Caribbean. Biologicals.

[8] Hamidi A., Boog C., Jadhav S., Kreeftenberg H. (2014). Lessons learned during the development and transfer of technology related to a new Hib conjugate vaccine to emerging vaccine manufacturers. Vaccine.

[9] Bozeman B. (2000). Technology transfer and public policy: A review of research and theory. Res. Policy.

[10] Bozeman B., Rimes H., Youtie J. (2015). The evolving state-of-the-art in technology transfer research: Revisiting the contingent effectiveness model. Res. Policy.

[11] Hendriks J. (2012). Technology transfer in human vaccinology: A retrospective review on public sector contributions in a privatizing science field. Vaccine.

[12] Clinton Health Access Initiative A Database of Local Vaccine Manufacturing Commitments and Tech-Transfers. https://www.clintonhealthaccess.org/database/a-database-of-local-vaccine-manufacturing-commitments-and-tech-transfers/.

[13] Medicines Patent Pool mRNA-Based Vaccine Initiatives—Up to May 2025. https://medicinespatentpool.org/what-we-do/mrna-technology-transfer-programme/resources#pills-Technical-Resources.

[14] Seventy-Eighth World Health Assembly WHA78.1 WHO Pandemic Agreement. https://apps.who.int/gb/ebwha/pdf_files/WHA78/A78_R1-en.pdf.

[15] Fifty-Eighth World Health Assembly WHA58.5 Strengthening Pandemic Influenza Preparedness and Response. https://apps.who.int/gb/ebwha/pdf_files/WHA58-REC1/english/A58_2005_REC1-en.pdf.

[16] Kieny M.P., Costa A., Hombach J., Carrasco P., Pervikov Y., Salisbury D., Greco M., Gust I., LaForce M., Franco-Paredes C. (2006). A global pandemic influenza vaccine action plan. Vaccine.

[17] Friede M., Palkonyay L., Alfonso C., Pervikov Y., Torelli G., Wood D., Kieny M.P. (2011). WHO initiative to increase global and equitable access to influenza vaccine in the event of a pandemic: Supporting developing country production capacity through technology transfer. Vaccine.

[18] Grohmann G., Francis D.P., Sokhey J., Robertson J. (2016). Challenges and successes for the grantees and the Technical Advisory Group of WHO’s influenza vaccine technology transfer initiative. Vaccine.

[19] Chadwick C., Friede M., Moen A., Nannei C., Sparrow E. (2022). Technology transfer programme for influenza vaccines–Lessons from the past to inform the future. Vaccine.

[20] Hendriks J., Holleman M., de Boer O., de Jong P., Luytjes W. (2011). An international technology platform for influenza vaccines. Vaccine.

[21] Collin N., Dubois P.M. (2011). The Vaccine Formulation Laboratory: A platform for access to adjuvants. Vaccine.

[22] Tarbet E.B., Dorward J.T., Day C.W., Rashid K.A. (2013). Vaccine production training to develop the workforce of foreign institutions supported by the BARDA influenza vaccine capacity building program. Vaccine.

[23] Ruiz J., Gilleskie G.L., Brown P., Burnett B., Carbonell R.G. (2014). Comprehensive hands-on training for influenza vaccine manufacturing: A WHO–BARDA–BTEC partnership for global workforce development. Biochem. Mol. Biol. Educ..

[24] Francis D.P., Grohmann G. (2011). WHO influenza vaccine technology transfer initiative: Role and activities of the Technical Advisory Group. Vaccine.

[25] Neuzil K.M., Tsvetnitsky V., Nyari L.J., Bright R.A., Boslego J.W., PATH’s Influenza Vaccine Project Team (2012). PATH Influenza Vaccine Project: Accelerating the development of new influenza vaccines for low-resource countries. Expert Rev. Vaccines.

[26] National Academies of Sciences Engineering and Medicine (2022). Countering the Pandemic Threat Through Global Coordination on Vaccines: The Influenza Imperative.

[27] World Health Organization (2021). Pandemic Influenza Preparedness Framework for the Sharing of Influenza Viruses and Access to Vaccines and Other Benefits.

[28] World Health Organization Category A: SMTA2 and Other Agreements with Vaccines & Antiviral Manufacturers. https://cdn.who.int/media/docs/default-source/pip-framework/smta2/20241025-smta2-benefits-cat-a.pdf?sfvrsn=17bc74dd_4.

[29] Taaffe J., Ostrowsky T.J., Mott J., Goldin S., Friede M., Gsell P., Chadwick C. (2024). Advancing influenza vaccines: A review of next-generation candidates and their potential for global health impact. Vaccine.

[30] Center for Infectious Disease Research and Policy Universal Influenza Vaccine Technology Landscape. https://ivr.cidrap.umn.edu/universal-influenza-vaccine-technology-landscape.

[31] Taaffe J., Goldin S., Lambach P., Sparrow E. (2025). Global production capacity of seasonal and pandemic influenza vaccines in 2023. Vaccine.

[32] Creswell J.W., Clark V.L.P. (2017). Designing and Conducting Mixed Methods Research.

[33] Sparrow E., Wood J.G., Chadwick C., Newall A.T., Torvaldsen S., Moen A., Torelli G. (2021). Global production capacity of seasonal and pandemic influenza vaccines in 2019. Vaccine.

[34] Hayman B., Pagliusi S. (2020). Emerging vaccine manufacturers are innovating for the next decade. Vaccine X.

[35] Scorza F.B. (2017). Advancing new vaccines against pandemic influenza in low-resource countries. Vaccine.

[36] Perdue M.L., Bright R.A. (2011). United States of America Department of Health and Human Services support for advancing influenza vaccine manufacturing in the developing world. Vaccine.

[37] International Federation of Pharmaceutical Manufacturers & Associations Technology Transfer: A Collaborative Approach to Improve Global Health. https://www.ifpma.org/publications/technology-transfer-a-collaborative-approach-to-improve-global-health/.

[38] Kumraj G., Pathak S., Shah S., Majumder P., Jain J., Bhati D., Hanif S., Mukherjee S., Ahmed S. (2022). Capacity Building for Vaccine Manufacturing Across Developing Countries: The Way Forward. Hum. Vaccines Immunother..

[39] Cole R. (2024). Inter-rater reliability methods in qualitative case study research. Sociol. Methods Res..

[40] Fetters M.D., Curry L.A., Creswell J.W. (2013). Achieving integration in mixed methods designs-principles and practices. Health Serv. Res..

[41] World Health Organization (2011). Report of the Fourth Meeting with International Partners on Prospects for Influenza Vaccine Technology Transfer to Developing Country Vaccine Manufacturers, Cancun, Mexico, 4–5 May 2011.

[42] Ponce-de-Leon S., Velazquez-Fernandez R., Bugarin-González J., García-Bañuelos P., Lopez-Sotelo A., Jimenez-Corona M.-E., Padilla-Catalan F., Cervantes-Rosales R. (2011). Domestic influenza vaccine production in Mexico: A state-owned and a multinational company working together for public health. Vaccine.

[43] World Health Organization (2016). Technical Report of Consultations with the Mexican Secretary of Health on Key Elements of Sustainability for Local Production of Influenza Vaccine Within the Context of Global Pandemic Preparedness.

[44] Palkonyay L., Fatima H. (2016). A decade of adaptation: Regulatory contributions of the World Health Organization to the Global Action Plan for Influenza Vaccines (2006–2016). Vaccine.

[45] World Health Organization (2010). Meeting with International Partners on Influenza Vaccine Production Technology Transfer to Developing Countries, 5–6 May 2010, Nha Trang, Vietnam.

[46] Stavaru C., Onu A., Lupulescu E., Tucureanu C., Rasid O., Vlase E., Coman C., Caras I., Ghiorghisor A., Berbecila L. (2016). Technology transfer of oil-in-water emulsion adjuvant manufacturing for pandemic influenza vaccine production in Romania: Preclinical evaluation of split virion inactivated H5N1 vaccine with adjuvant. Hum. Vaccines Immunother..

[47] Blume S., Baylac-Paouly B. (2021). Immunization and States: The Politics of Making Vaccines.

[48] Latin-American Institute of Biotechnology Mechnikov. https://mechnikov.com/en/main.

[49] World Health Organization (2015). Report of the Eighth Meeting with International Partners on Prospects for Influenza Vaccine Technology Transfer to Developing Country Vaccine Manufacturers: Sao Paulo, Brazil, 17–18 March 2015.

[50] World Health Organization WHO/UNICEF Joint Reporting Form on Immunization: Influenza Vaccination Policy. https://immunizationdata.who.int/global/wiise-detail-page/influenza-vaccination-policy.

[51] World Health Organization WHO Public Inspection Report: Instituto Latinoamericano de Biotecnologia Mechnikov SA. https://extranet.who.int/prequal/sites/default/files/whopir_files/WHOPIR_Mechnikov_24-28April2023.pdf.

[52] World Health Organization (2008). Meeting with International Partners on Prospects for Influenza Vaccine Technology Transfer to Developing Countries, 27–28 November 2008, Pune, Maharashtra, India.

[53] Friede M., Serdobova I., Palkonyay L., Kieny M.P. (2009). Technology transfer hub for pandemic influenza vaccine. Vaccine.

[54] Hendriks J., Holleman M., Hamidi A., Beurret M., Boog C. (2013). Vaccinology capacity building in Europe for innovative platforms serving emerging markets. Hum. Vaccines Immunother..

[55] World Health Organization (2012). Report of the Fifth Meeting with International Partners on Prospects for Influenza Vaccine Technology Transfer to Developing Country Vaccine Manufacturers, Belgrade, Serbia, 27–28 March 2012.

[56] Sarsenbayeva G., Volgin Y., Kassenov M., Issagulov T., Bogdanov N., Sansyzbay A., Abitay R., Nurpeisova A., Sagymbay A., Koshemetov Z. (2018). Safety and immunogenicity of the novel seasonal preservative-and adjuvant-free influenza vaccine: Blind, randomized, and placebo-controlled trial. J. Med. Virol..

[57] Sarsenbayeva G., Volgin Y., Kassenov M., Issagulov T., Bogdanov N., Sansyzbay A., Stukova M., Buzitskaya Z., Kulmagambetov I., Davlyatshin T. (2018). Immunogenicity and safety of a novel seasonal influenza preservative-free vaccine manufactured in Kazakhstan: Results of a randomized, comparative, phase II clinical trial in adults. Hum. Vaccines Immunother..

[58] Sarsenbayeva G., Issagulov T., Kassenov M., Abitay R., Orynbayev M., Stukova M., Pisareva M., Davlyatshin T., Lespek K., Khairullin B. (2020). Safety and immunogenicity of trivalent inactivated influenza vaccine in adults 60 years of age and older: A phase II, a randomized, comparative trial in Kazakhstan. Hum. Vaccines Immunother..

[59] Jadhav S., Dhere R., Yeolekar L., Gautam M. (2010). Influenza Vaccine Production Capacity Building in Developing Countries: Example of the Serum Institute of India. Procedia Vaccinol..

[60] Dhere R., Yeolekar L., Kulkarni P., Menon R., Vaidya V., Ganguly M., Tyagi P., Barde P., Jadhav S. (2011). A pandemic influenza vaccine in India: From strain to sale within 12 months. Vaccine.

[61] World Health Organization (2016). Report of the Ninth Meeting with International Partners on Prospects for Influenza Vaccine Technology Transfer to Developing Country Vaccine Manufacturers: Geneva, Switzerland, 17 November 2016.

[62] Adbi A., Chatterjee C., Drev M., Mishra A. (2019). When the Big One Came: A Natural Experiment on Demand Shock and Market Structure in India’s Influenza Vaccine Markets. Prod. Oper. Manag..

[63] Egypt National Strategy for Vaccine Manufacturing Localization 2024–2030. https://upa.gov.eg/wp-content/uploads/2025/06/National_Vaccine_Manufacturing_Localization_Strategy.pdf.

[64] Nannei C., Goldin S., Torelli G., Fatima H., Kumar K., Bubb-Humfryes O., Stenson B., Sparrow E. (2016). Stakeholders’ perceptions of 10 years of the Global Action Plan for Influenza Vaccines (GAP)–Results from a survey. Vaccine.

[65] Dutt D., Mazzucato M., Torreele E. (2024). An mRNA technology transfer programme and economic sustainability in health care. Bull. World Health Organ..

[66] Cooper L.A., Brennan C.M., Leslie D.G., Brown A.J. (2024). Integrating Literature as a Data Source in Mixed Methods Research. J. Mix. Methods Res..

[67] Onwuegbuzie A.J., Collins K.M. (2007). A typology of mixed methods sampling designs in social science research. Qual. Rep..

