# Supplementary material for: Influenza Vaccine Technology Transfer: A Mixed-Methods Study with Vaccine Manufacturers and Global Experts to Assess Successes, Challenges, and Opportunities
Source: Vaccines (Basel). 2026 Jun 11;14(6):522. doi: 10.3390/vaccines14060522 (PMC13308326; doi:10.3390/vaccines14060522)
Supplement: Supplementary file 1 [file vaccines-14-00522-s001.zip › vaccines-4292435-supplementary.pdf]

Supplementary materials

Survey instruments

S1. Local/national manufacturers survey (p. 2)
S2. Multinational manufacturers survey (p. 10)
S3. Experts survey (p. 13)

Interview guides

S4. Manufacturers interview guide (p. 16)
S5. Experts interview guide (p. 17)

Qualitative coding framework

S6. Revised codebook for interview analysis (p. 18)

Supporting data

S7. Summary table of IVTT observations (p. 22)
S8. Summary table of importance rating for TT enablers (p. 23)
S9. Summary table of weighted ranks for TT enablers (p. 24)
S.10 Additional IVTT recipients and supplier (p.25)

References

S11. References for supplementary materials (p. 29)

S1. Local/national manufacturers survey

Survey focal point

The following information will be collected to identify the survey focal point. This information will only be used if we need to contact you for any follow-up questions.

1. **Contact information**
   Focal point name:
   Focal point email:
   Company/organization name:
   Country:

Manufacturer characteristics

This section aims to collection basic characteristics regarding your company.

1. **Please identify the management structure of your company.**
   - ☐ Non-profit
   - ☐ Private
   - ☐ State/government owned
   - ☐ Other – *Please specify:*

2. **What is the overall product landscape in your company?** *Please indicate the number of influenza vaccines (both seasonal and pandemic/pre-pandemic) and other vaccines (not including influenza) that are approved by a national regulatory authority (NRA) and WHO prequalified. Note that pandemic influenza vaccine would include the 2009 A/H1N1 vaccine and pre-pandemic influenza vaccine could include those for A/H2N2, A/H5N1, A/H7N9, etc. Also, note that an approved pre-pandemic influenza vaccine would include those approved through a mock dossier by a national regulatory authority.*

Product	Approved by NRA	WHO prequalified
Seasonal influenza vaccines		
Monovalent pandemic/pre-pandemic influenza vaccines		
Other vaccines (not including influenza)		

3. **Please estimate the annual installed production capacity (in total doses) for all vaccines produced by your company.**
   - ☐ <5 million doses/year
   - ☐ 5-25 million doses/year
   - ☐ 26-100 million doses/year
   - ☐ >100 million doses/year

4. **For the most recent influenza seasons, what <u>percentage</u> of your seasonal influenza vaccine production output was supplied to the public and private markets?** *If your company no longer produces an influenza vaccine or doesn't produce one for a certain hemisphere, please write "N/A."*

Influenza season	Public market	Private market
2022-23 Northern Hemisphere Season		
2022 Southern Hemisphere Season		

5. **Do other vaccine manufacturers supply seasonal influenza vaccines in your country?**
   - ☐ Yes
   - ☐ No
   - ☐ Unsure

6. **Does your company have a procurement agreement with your country's government for the influenza vaccine?** *Please note that this would apply to both seasonal influenza vaccines (annual or multi-year procurement) and pandemic influenza vaccines (advance purchase agreement). Please check all that apply.*
   - ☐ Yes, for procurement of seasonal influenza vaccines
   - ☐ Yes, for advance purchase of pandemic influenza vaccines
   - ☐ No
7. **Does your company supply a seasonal influenza vaccine to a regional or global procurement mechanism (e.g., the PAHO Revolving Fund, UNICEF)?**
   - ☐ Yes
   - ☐ No

Technology transfer effectiveness

Please note that this section of the survey should be completed by manufacturers that indicated in question 1 in the previous section that they previously participated in a technology transfer agreement for influenza vaccines.

1. **Prior to the relevant influenza vaccine technology transfer, did your company have previous experience as a technology <u>supplier</u> in a technology transfer agreement for any type of vaccine?**
   - ☐ Yes
   - ☐ No

2. **Prior to the relevant influenza vaccine technology transfer, did your company have previous experience as a technology <u>recipient</u> in a technology transfer agreement for any type of vaccine?**
   - ☐ Yes
   - ☐ No

3. **Prior to the influenza vaccine technology transfer, did your company ever produce influenza vaccines?** *Even if your company no longer produces the influenza vaccine, please select yes.*
   - ☐ Yes
   - ☐ No

4. **Prior to the influenza vaccine technology transfer, did your country have a national seasonal influenza vaccination policy or were seasonal influenza vaccines used in the country?**
   - ☐ Yes
   - ☐ No
   - ☐ Unknown

5. **Which types of influenza vaccines has your company pursued through technology transfer?** *Please check all that apply. If your company pursued multiple technology transfer agreements for the same type of influenza vaccine, please select "Additional influenza vaccine".*
   - ☐ Seasonal inactivated influenza vaccine, EGG-based
   - ☐ Seasonal inactivated influenza vaccine, CELL-based
   - ☐ Seasonal live attenuated influenza vaccine, EGG-based
   - ☐ Seasonal live attenuated influenza vaccine, CELL-based
   - ☐ Seasonal recombinant influenza vaccine
   - ☐ Pre-pandemic inactivated influenza vaccine, EGG-based
   - ☐ Pre-pandemic inactivated influenza vaccine, CELL-based
   - ☐ Pre-pandemic live attenuated influenza vaccine, EGG-based
   - ☐ Pre-pandemic live attenuated influenza vaccine, CELL-based
   - ☐ Other influenza vaccine – *Please specify:*
   - ☐ Additional influenza vaccine – *Please specify:*
   - ☐ Not applicable (only participated as technology transfer supplier) *(proceed to next section – F. Enablers of technology transfer)*

The questions in the following section should be answered for each influenza vaccine type identified in question 5.

6. **Through what mechanism did your company receive the influenza vaccine technology?**
   - ☐ Bilateral agreement
   - ☐ WHO technology transfer initiative
   - ☐ Other – *Please specify:*

7. **What was the method for the transfer of technology?** *Please refer to the following figure for an overview of each method.*

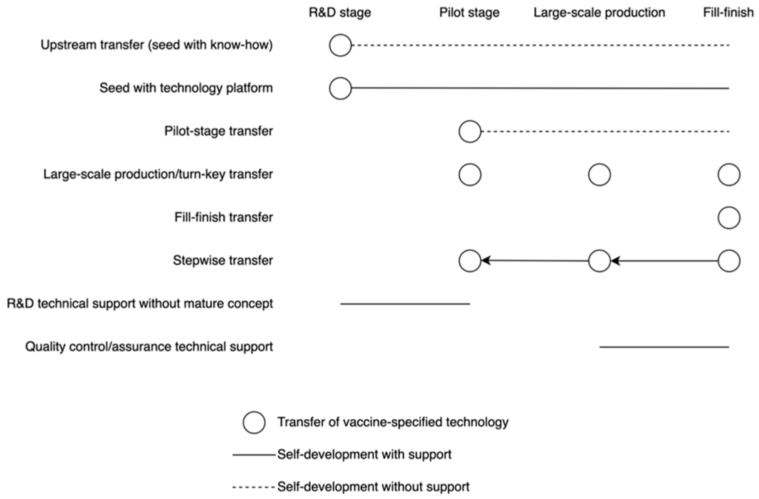

☐ Upstream transfer (seed with know-how)
☐ Seed with technology platform
☐ Pilot stage transfer
☐ Large-scale production/turnkey transfer
☐ Fill-finish transfer
☐ Stepwise transfer
☐ R&D technical support
☐ Quality assurance/quality control technical support
☐ Other – *Please specify:*

8. **What was your company's motivation for pursuing influenza vaccine technology transfer?** *Please check all that apply.*

☐ Approached by or had previous experience with technology supplier
☐ Encouraged by government
☐ Expand technology capacity/capability
☐ Reestablish/update influenza vaccine production
☐ Strengthen epidemic and pandemic preparedness
☐ Other – *Please specify:*

9. **When did your company initiate the influenza vaccine technology transfer agreement?** *Please indicate the YEAR that the agreement was initiated.*

10. **Did your country's government provide direct financial support for the influenza vaccine technology transfer?**

☐ Yes
☐ No

11. **What is the status of the technology transfer?**
    - ☐ Completed *(proceed to question 12)*
    - ☐ Ongoing *(proceed to next section – F. Enablers of technology transfer)*
    - ☐ Planned *(proceed to next section – F. Enablers of technology transfer)*
    - ☐ Terminated before completion *(proceed to question 12)*

12. **When was the technology transfer completed or terminated?** *Please indicate the YEAR.*

Questions 13-24 apply to the actual influenza vaccine that resulted from the technology transfer that was completed.

13. **Did the technology transfer result in an approved influenza vaccine?**
    - ☐ Yes *(proceed to question 14)*
    - ☐ No *(proceed to question 15)*

14. **When was the influenza vaccine approved?** *Please indicate the YEAR that the influenza vaccine resulting from the technology transfer was approved by the national regulatory authority. For a pre-pandemic influenza vaccine, this would be when the mock dossier was approved by the national regulatory authority.*

15. **Is the vaccine still produced?** *Please note that this question applies to seasonal influenza vaccines that have resulted from the technology transfer.*
    - ☐ Yes
    - ☐ No
    - ☐ Not applicable

16. **Was the associated intellectual property transferred along with the know-how for the influenza vaccine?**
    - ☐ Yes
    - ☐ No
    - ☐ Not applicable

17. **Is the influenza vaccine exported?** *Please note that this question applies to seasonal influenza vaccines that have resulted from the technology transfer.*
    - ☐ Yes
    - ☐ No
    - ☐ Not applicable

18. **Did staff in your company receive relevant influenza vaccine production training from the technology supplier?**
    - ☐ Yes
    - ☐ No

19. **Were the skills and know-how obtained through influenza vaccine technology transfer leveraged for the development and production of other vaccines?** *Please note that this question refers to the skills and know-how, NOT facilities or equipment.*

**Vaccine**	**Yes**	**No**
Other influenza vaccine	☐	☐
COVID-19 vaccine	☐	☐
Routine/EPI vaccine	☐	☐
Other vaccine	☐	☐

20. **Were the facilities and equipment established through the influenza vaccine technology transfer leveraged for the development and production of other vaccines?** *Please note that this question refers to the facilities or equipment, NOT skills and know-how.*

**Vaccine**	**Yes**	**No**
Other influenza vaccine	☐	☐
COVID-19 vaccine	☐	☐
Routine/EPI vaccine	☐	☐
Other vaccine	☐	☐

21. **Has your country's government provided direct or indirect financial support to your company following the completion of the influenza vaccine technology transfer (e.g., through subsidies)?**

    ☐ Yes
    ☐ No

22. **Does your company have a procurement agreement with your country's government for the influenza vaccine?** *For example, annual procurement for seasonal influenza vaccines or advance purchase agreement for pandemic influenza vaccines.*

    ☐ Yes
    ☐ No

23. **Does your company supply the influenza vaccine to a regional or global procurement mechanism (e.g., the PAHO Revolving Fund, UNICEF)?** *Please note that this question applies to seasonal influenza vaccines that have resulted from the technology transfer.*

    ☐ Yes
    ☐ No

24. **What is the maximum production capacities for the influenza vaccine resulting from the technology transfer?** *Please estimate the capacity in terms of maximum theoretical capacity (in number of doses) that could be produced in 12 months.*

Enablers of technology transfer

1. **Based on the influenza vaccine technology transfer experience, please review each factor and indicate its level of importance as an enabler for technology transfer.**
   Please indicate the level of importance according to the following scale:
   - *1: Not at all important*
   - *2: Relatively unimportant*
   - *3: Neutral – neither important nor unimportant*
   - *4: Moderately important*
   - *5: Critically important*
   - *N/A: Not applicable*

**Enablers**	**Level of Importance**					
	**(1)**	**(2)**	**(3)**	**(4)**	**(5)**	**N/A**
Adherence to ethical standards	☐	☐	☐	☐	☐	☐
Adherence to regulatory standards	☐	☐	☐	☐	☐	☐
Access to appropriate capital markets (i.e., project funding)	☐	☐	☐	☐	☐	☐
Business development experience	☐	☐	☐	☐	☐	☐
Clear economic development priorities	☐	☐	☐	☐	☐	☐
Common values, trust, and commitment from all parties	☐	☐	☐	☐	☐	☐
Experience with evaluating the quality of technologies	☐	☐	☐	☐	☐	☐
Government commitment to local production and procurement of vaccine	☐	☐	☐	☐	☐	☐
Functional national regulatory authority	☐	☐	☐	☐	☐	☐
Innovation-friendly environment with sound intellectual property rights	☐	☐	☐	☐	☐	☐
Political stability and transparent governance	☐	☐	☐	☐	☐	☐
Proper access to information (e.g., available resources, market opportunities)	☐	☐	☐	☐	☐	☐
Resource prioritization at the company level	☐	☐	☐	☐	☐	☐
Rule of Law/legal system is established and enforced	☐	☐	☐	☐	☐	☐
R&D capacity of the technology recipient	☐	☐	☐	☐	☐	☐
Viable and accessible local market	☐	☐	☐	☐	☐	☐
Workforce recruitment, training, retention	☐	☐	☐	☐	☐	☐

2. **Of the enablers identified in the previous question, please identify and rank the top 5 enablers as contributing to the effectiveness of influenza vaccine technology transfer.**
   Note that all enablers from the previous question should be taken into consideration; they are listed below in alphabetical order. Of the 5 factors that are identified, please rank them 1-5, with 1 being the most impactful.

Enablers	Ranking
Adherence to ethical standards	
Adherence to regulatory standards	
Access to appropriate capital markets (i.e., project funding)	
Business development experience	
Clear economic development priorities	
Common values, trust, and commitment from all parties	
Experience with evaluating the quality of technologies	
Government commitment to local production and procurement of vaccine	
Functional national regulatory authority	
Innovation-friendly environment with sound intellectual property rights	
Political stability and transparent governance	
Proper access to information (e.g., available resources, market opportunities)	
Resource prioritization at the company level	
Rule of Law/legal system is established and enforced	
R&D capacity of the technology recipient	
Viable and accessible local market	
Workforce recruitment, training, retention	

3. **Are there other factors that you would consider as enablers for influenza vaccine technology transfer?**

Interview

1. **Do you agree to participate in a follow up interview to discuss your influenza vaccine technology transfer experience in more detail?** *Please note that the interview will be conducted virtually and should take no more than 45 minutes. If you agree to participate, the researcher will follow up to schedule the interview.*

   ☐ Yes
   ☐ No

S2. Multinational manufacturers survey

Survey focal point

The following information will be collected to identify the survey focal point. This information will only be used if we need to contact you for any follow-up questions.

1. **Contact information**
   Focal point name:
   Focal point email:
   Company/organization name:
   Country:

Manufacturer characteristics

This section aims to collection basic characteristics regarding your company.

1. **Has your company participated in a technology transfer agreement as a supplier of influenza vaccine production technology?**
   ☐ Yes *(go to question 2)*
   ☐ No *(end of survey)*

2. **How many influenza vaccine technology transfer agreements has your company conducted?** *Please note that this includes agreements that have already been initiated but might not be completed.*

3. **Which manufacturer(s) received the influenza vaccine technology?**

Enablers of technology transfer

1. **Based on the influenza vaccine technology transfer experience, please review each factor and indicate its level of importance as an enabler for technology transfer.**
   Please indicate the level of importance according to the following scale:
   - *1: Not at all important*
   - *2: Relatively unimportant*
   - *3: Neutral – neither important nor unimportant*
   - *4: Moderately important*
   - *5: Critically important*
   - *N/A: Not applicable*

Enablers	Level of Importance					
	(1)	(2)	(3)	(4)	(5)	N/A
Adherence to ethical standards	☐	☐	☐	☐	☐	☐
Adherence to regulatory standards	☐	☐	☐	☐	☐	☐
Access to appropriate capital markets (i.e., project funding)	☐	☐	☐	☐	☐	☐

Business development experience	☐	☐	☐	☐	☐	☐
Clear economic development priorities	☐	☐	☐	☐	☐	☐
Common values, trust, and commitment from all parties	☐	☐	☐	☐	☐	☐
Experience with evaluating the quality of technologies	☐	☐	☐	☐	☐	☐
Government commitment to local production and procurement of vaccine	☐	☐	☐	☐	☐	☐
Functional national regulatory authority	☐	☐	☐	☐	☐	☐
Innovation-friendly environment with sound intellectual property rights	☐	☐	☐	☐	☐	☐
Political stability and transparent governance	☐	☐	☐	☐	☐	☐
Proper access to information (e.g., available resources, market opportunities)	☐	☐	☐	☐	☐	☐
Resource prioritization at the company level	☐	☐	☐	☐	☐	☐
Rule of Law/legal system is established and enforced	☐	☐	☐	☐	☐	☐
R&D capacity of the technology recipient	☐	☐	☐	☐	☐	☐
Viable and accessible local market	☐	☐	☐	☐	☐	☐
Workforce recruitment, training, retention	☐	☐	☐	☐	☐	☐

2. **Of the enablers identified in the previous question, please identify and rank the top 5 enablers as contributing to the effectiveness of influenza vaccine technology transfer.** *Note that all enablers from the previous question should be taken into consideration; they are listed below in alphabetical order. Of the 5 factors that are identified, please rank them 1-5, with 1 being the most impactful.*

Enablers	Ranking
Adherence to ethical standards	
Adherence to regulatory standards	
Access to appropriate capital markets (i.e., project funding)	
Business development experience	
Clear economic development priorities	
Common values, trust, and commitment from all parties	
Experience with evaluating the quality of technologies	
Government commitment to local production and procurement of vaccine	
Functional national regulatory authority	
Innovation-friendly environment with sound intellectual property rights	
Political stability and transparent governance	
Proper access to information (e.g., available resources, market opportunities)	
Resource prioritization at the company level	
Rule of Law/legal system is established and enforced	
R&D capacity of the technology recipient	
Viable and accessible local market	

Workforce recruitment, training, retention	

3. **Are there other factors that you would consider as enablers for influenza vaccine technology transfer?**

Interview

1. **Do you agree to participate in a follow up interview to discuss your influenza vaccine technology transfer experience in more detail?** *Please note that the interview will be conducted virtually and should take no more than 45 minutes. If you agree to participate, the researcher will follow up to schedule the interview.*
   - ☐ Yes
   - ☐ No

S3. Experts survey

Survey focal point

The following information will be collected to identify the survey focal point. This information will only be used if we need to contact you for any follow-up questions.

1. **Contact information**
   Focal point name:
   Focal point email:
   Country:

Expert characteristics

1. **Please identify your primary area of expertise with respect to vaccines.**
   - ☐ Business development
   - ☐ Manufacturing
   - ☐ Regulatory issues
   - ☐ Research and development
   - ☐ Other – Please specify:

2. **Were you a member of the Technical Advisory Group as part of the WHO Technology Transfer Initiative for influenza vaccines?**
   - ☐ Yes
   - ☐ No

3. **Have you provided direct technical assistance to influenza vaccine manufacturers as it pertains to technology transfer?** *For example, you worked directly with a manufacturer on their influenza vaccine technology transfer versus being part of an advisory group tasked with overseeing influenza vaccine technology transfer.*
   - ☐ Yes
   - ☐ No

Enablers of technology transfer

1. **Based on the influenza vaccine technology transfer experience, please review each factor and indicate its level of importance as an enabler for technology transfer.**
   Please indicate the level of importance according to the following scale:
   - *1: Not at all important*
   - *2: Relatively unimportant*
   - *3: Neutral – neither important nor unimportant*
   - *4: Moderately important*
   - *5: Critically important*
   - *N/A: Not applicable*

Enablers	Level of Importance					
	(1)	(2)	(3)	(4)	(5)	N/A
Adherence to ethical standards	☐	☐	☐	☐	☐	☐
Adherence to regulatory standards	☐	☐	☐	☐	☐	☐
Access to appropriate capital markets (i.e., project funding)	☐	☐	☐	☐	☐	☐
Business development experience	☐	☐	☐	☐	☐	☐
Clear economic development priorities	☐	☐	☐	☐	☐	☐
Common values, trust, and commitment from all parties	☐	☐	☐	☐	☐	☐
Experience with evaluating the quality of technologies	☐	☐	☐	☐	☐	☐
Government commitment to local production and procurement of vaccine	☐	☐	☐	☐	☐	☐
Functional national regulatory authority	☐	☐	☐	☐	☐	☐
Innovation-friendly environment with sound intellectual property rights	☐	☐	☐	☐	☐	☐
Political stability and transparent governance	☐	☐	☐	☐	☐	☐
Proper access to information (e.g., available resources, market opportunities)	☐	☐	☐	☐	☐	☐
Resource prioritization at the company level	☐	☐	☐	☐	☐	☐
Rule of Law/legal system is established and enforced	☐	☐	☐	☐	☐	☐
R&D capacity of the technology recipient	☐	☐	☐	☐	☐	☐
Viable and accessible local market	☐	☐	☐	☐	☐	☐
Workforce recruitment, training, retention	☐	☐	☐	☐	☐	☐

2. **Of the enablers identified in the previous question, please identify and rank the top 5 enablers as contributing to the effectiveness of influenza vaccine technology transfer.** *Note that all enablers from the previous question should be taken into consideration; they are listed below in alphabetical order. Of the 5 factors that are identified, please rank them 1-5, with 1 being the most impactful.*

Enablers	Ranking
Adherence to ethical standards	
Adherence to regulatory standards	
Access to appropriate capital markets (i.e., project funding)	
Business development experience	
Clear economic development priorities	
Common values, trust, and commitment from all parties	
Experience with evaluating the quality of technologies	
Government commitment to local production and procurement of vaccine	
Functional national regulatory authority	
Innovation-friendly environment with sound intellectual property rights	
Political stability and transparent governance	

Proper access to information (e.g., available resources, market opportunities)	
Resource prioritization at the company level	
Rule of Law/legal system is established and enforced	
R&D capacity of the technology recipient	
Viable and accessible local market	
Workforce recruitment, training, retention	

3. **Are there other factors that you would consider as enablers for influenza vaccine technology transfer?**

Interview

1. **Do you agree to participate in a follow up interview to discuss your influenza vaccine technology transfer experience in more detail?** *Please note that the interview will be conducted virtually and should take no more than 45 minutes. If you agree to participate, the researcher will follow up to schedule the interview.*
   - ☐ Yes
   - ☐ No

S4. Manufacturers interview guide

1. Please provide a brief overview of your experience with influenza vaccine technology transfer, including when you were involved and what your role was.
2. When looking at the contingent effectiveness model, it can be assumed that the five determinants collectively impact how effective technology transfer is. In your experience, how would you characterize the determinants, their interactions with each other, and their impact on the effectiveness of influenza vaccine tech transfer?
3. Which of these determinants, if any, do you think were most impactful to the effectiveness of the influenza vaccine technology transfer?
4. The model assesses effectiveness in terms of direct benefits, such as if the technology was actually transferred and if it resulted in an approved product, but it also recognizes non-material and spillover benefits, such as supporting other vaccine development, increasing workforce skills, enhancing political reward and recognition by the host government, and strengthening shared public values. Based on your experience with influenza vaccine technology transfer, please characterize its effectiveness in terms of these six criteria. What worked? What did not work?
5. In your experience, do the stakeholders you've worked with, including in your own organization, value non-material and spillover benefits as much as the direct benefits of influenza vaccine technology transfer?
6. Are there determinants or effectiveness criteria that are more relevant for influenza vaccine technology transfer versus technology transfer of other vaccines?
7. *(For GAP/TTI participants)* How would you characterize the influenza vaccine technology transfer initiative as part of the WHO Global Action Plan for Influenza Vaccines, or GAP? What worked? What did not work?
8. Based on your experience, can you provide more details on what you observed as the primary enablers for effective influenza vaccine technology transfer? For this question, we'll define effective technology transfer in terms of completion of the transfer agreement.
9. Of this list of enablers, are there any that you think are more relevant for influenza vaccine technology transfer versus technology transfer of other vaccines?
10. Are there enablers that are missing from this list?
11. Regarding next-generation influenza vaccines, do you have any recommendations for how technology transfer of those vaccines should be conducted?
12. Do you have any other comments or reflections that you would like to share?

S5. Experts interview guide

1. Please provide a brief overview of your experience with influenza vaccine technology transfer, including when you were involved and what your role was.
2. When looking at the contingent effectiveness model, it can be assumed that the five determinants collectively impact how effective technology transfer is. In your experience, how would you characterize the determinants, their interactions with each other, and their impact on the effectiveness of influenza vaccine tech transfer?
3. Which of these determinants, if any, do you think were most impactful to the effectiveness of the influenza vaccine technology transfer?
4. The model assesses effectiveness in terms of direct benefits, such as if the technology was actually transferred and if it resulted in an approved product, but it also recognizes non-material and spillover benefits, such as supporting other vaccine development, increasing workforce skills, enhancing political reward and recognition by the host government, and strengthening shared public values. Based on your experience with influenza vaccine technology transfer, please characterize its effectiveness in terms of these six criteria. What worked? What did not work?
5. In your experience, do you think vaccine manufacturers value non-material and spillover benefits as much as the direct benefits of influenza vaccine technology transfer?
6. Are there determinants or effectiveness criteria that are more relevant for influenza vaccine technology transfer versus technology transfer of other vaccines?
7. *(For GAP/TTI participants)* How would you characterize the influenza vaccine technology transfer initiative as part of the WHO Global Action Plan for Influenza Vaccines, or GAP? What worked? What did not work?
8. *(For GAP/TTI participants)* If given the opportunity and knowledge gained, would you have designed GAP differently? If so, how?
9. Based on your experience, can you provide more details on what you observed as the primary enablers for effective influenza vaccine technology transfer? For this question, we'll define effective technology transfer in terms of completion of the transfer agreement.
10. Of this list of enablers, are there any that you think are more relevant for influenza vaccine technology transfer versus technology transfer of other vaccines?
11. Are there enablers that are missing from this list?
12. Regarding next-generation influenza vaccines, do you have any recommendations for how technology transfer of those vaccines should be conducted?
13. Do you have any other comments or reflections that you would like to share?

S6. Revised codebook for interview analysis

Code	Sub-codes	Definition	Coding criteria	Revision notes
Transfer recipient	• Business development<br>• Ethical standards<br>• Experience with TT<br>• Experience with vaccine production<br>• Regulatory standards<br>• Resource prioritization<br>• R&D capacity<br>• Technology assessment	The institution receiving the influenza vaccine production technology, with emphasis on internal capacities, experience, governance, and institutional readiness	Include: project management, negotiation skills, ethical standards within the institution, compliance with national and international regulatory standards and norms, internal allocation of resources (staff, funding, time, infrastructure) toward TT, scientific and R&D capacity prior to TT, ability to evaluate suitability of technology, past experience with manufacturing vaccines<br><br>Exclude: national-level rule of law, supplier-led processes, R&D capacity/capabilities post-TT	New "experience with vaccine production" sub-code proposed to emphasize operational experience versus R&D experience
Transfer supplier	• Business development<br>• Ethical standards<br>• Experience with TT<br>• Regulatory standards	Characteristics, behaviors, capacities, and engagement of the technology provider	Include: references to the role, support, transparency, or engagement from the supplier	New "experience with TT" sub-code proposed to capture how prior TT experience shapes the supplier's approach with the relevant IVTT
Transfer object	• IP & innovation	The influenza vaccine production technology that is being transferred, including production platform, process, complexity, and required inputs	Include: seasonal or pre-pandemic/pandemic application, technology platform – IIV, LAIV, RIV; technology substrate – eggs, cells; consumables and inputs; IP related to the vaccine/process	Clarified so that the code relates to the technology itself and not demand or institutional constraints, non-specific TT methods and processes, or seasonal/pandemic motivations for TT

			Exclude: non-influenza vaccines, NGIV recommendations, TT methods and processes not specific to influenza, seasonal/pandemic motivations for TT	
Transfer medium	• Trust & commitment	The mechanism, modality, and relational dynamics through which IVTT occurs	Include: mutual trust, transparency, continuity in recipient-supplier relationship; TT mechanism – bilateral agreements, hubs, joint ventures; TT process steps – training, standard operating procedures, technical assistance/scale-up support; TT method – fill/finish, large-scale production, pilot stage, etc.<br><br>Exclude: transfer recipient/supplier capabilities	Clarified that this code is relational or process-based versus individual institutional capabilities that would fall under "transfer recipient" or "transfer supplier"
Demand environment	• Economic development priorities<br>• Functional NRA<br>• Government commitment<br>• Political stability	External conditions shaping motivation and feasibility of IVTT, including market, policy, regulatory, and political contexts	Include: national industrial or development goals motivating IVTT; capacity of the NRA; government procurement, policy support, and political will, including financial support for TT; political stability at the national level; public trust in vaccines; market considerations prior to TT<br><br>Exclude: recipient/supplier level stability; market considerations post-TT	Clarified misclassifications of company versus national stability and pre- versus post-TT vaccine market considerations

Cross-cutting enablers	• Access to capital<br>• Access to information<br>• Rule of law<br>• Vaccine market<br>• Workforce	IVTT enablers covering themes that cross institutional, process, and systemic levels	Include: funding availability, donor support, market intelligence, demand forecasts, national legal infrastructure and impact on contracting, workforce availability and national policies<br><br>Exclude: ethical standards, workforce training associated with IVTT	New parent code created for enablers that apply to recipients, suppliers, government, and/or regulators
Out-the-door	N/A	An institution has received the influenza vaccine technology and/or training from the supplier	Include: signed agreements, initial technology receipt or training, preclinical and clinical development<br><br>Exclude: considerations of impact, vaccine approval	Added preclinical/clinical development that could be a signal for successful out-the-door transfer
Market impact & economic development	N/A	Commercial impact of the IVTT, including approval of the vaccine and national/regional impact beyond the institution	Include: product licensure, revenue, commercial success of the IVTT, sustainability<br><br>Exclude: business motivations for IVTT	Added long-term sustainability
Political advantage	N/A	Political reward in the form of production subsidies, procurement preferences, political visibility, and strengthened manufacturer-government/NRA relationships	Include: political visibility, strengthened manufacturer-government/NRA relationships, political support or assistance following IVTT	No changes
Human capital	N/A	Staff trained as part of the IVTT	Include: human resource capacities, staff retention, training outcomes	Clarifications of workforce to differentiate workforce/training items between transfer media, cross-cutting enablers, and human capital

			Exclude: national level workforce policies and impact, training modalities	
Opportunity costs	N/A	Impact of the IVTT, including know-how and facilities, on the development of other vaccines or future TTs	Include: projects paused or deprioritized, shifts in focus, facility/technology use for other vaccines, TT experience enabling future TT capacity<br><br>Exclude: consumables and supplies	Clarified to exclude consumables and supplies which now fall under transfer object
Public value	N/A	Impact of the IVTT on preparedness, equitable access, national stockpiling, and pandemic readiness	Include: national, regional, and global vaccine access, role of recipient in pandemic planning, production capacity,<br><br>Exclude: epidemic/pandemic preparedness motivation for IVTT	Clarified to exclude epidemic and pandemic preparedness as a motivation for IVTT

S7. Summary table of IVTT observations

TT status[1]	IVTT role[2]	Management structure	Technology & substrate	Application	Mechanism	Method	Government support prior to IVTT	Vaccine approved	TT start (year)	TT completion/ termination (year)	Vaccine approved (year)	IP transferred	Vaccine exported	Government support after IVTT completion	Procurement agreement with government	Regional procurement
C	R	Public	IIV-egg	Seasonal	Bilateral	Fill/finish	No	Yes	2008	2008	2009	Yes	No	No	No	No
C	R	Public	LAIV-egg	Pandemic	Multilateral	Pilot stage	No	Yes	2009	2009	2010	No	N/A[3]	ND[4]	ND	N/A
C	R	Private	RIV-cell	Seasonal	Bilateral	Pilot stage	No	Yes	2009	2010	2016	No	No	No	No	No
C	R	Private	RIV-cell	Pandemic	Bilateral	Pilot stage	No	Yes	2009	2010	2015	No	N/A	No	No	N/A
C	R	Private	IIV-egg	Pandemic	Bilateral	Fill/finish	No	Yes	2010	2011	2011	No	N/A	No	No	N/A
C	R	Private	IIV-egg	Seasonal	Bilateral	Fill/finish	No	Yes	2010	2013	2013	No	Yes	No	Yes	Yes
C	R	Private	a-IIV-egg	Seasonal	Bilateral	Fill/finish	No	Yes	2012	2014	2015	No	No	No	Yes	No
C	R	Public	IIV-egg	Seasonal	Bilateral	Large-scale	No	Yes	1999	2014	2012	Yes	Yes	Yes	Yes	Yes
C	R	Private	IIV-egg	Seasonal	Bilateral	Fill/finish	No	Yes	2015	2015	2015	No	Yes	No	No	No
C	R	Private	LAIV-egg	Seasonal	Multilateral	Pilot stage	No	Yes	2011	2016	2020	Yes	No	No	No	No
C	R	Public	IIV-egg	Seasonal	Bilateral	R&D tech support	No	No	2009	2016	N/A	No	N/A	Yes	No	N/A
C	R	Public	IIV-egg	Seasonal	Multilateral	Large-scale	No	Yes	2011	2018	2019	Yes	No	No	No	No
C	R	Public	IIV-egg	Seasonal	Multilateral	Seed w/tech platform	Yes	Yes	2009	2018	2020	No	No	No	No	No
C	R	Public	IIV-egg	Pandemic	Multilateral	Seed w/tech platform	Yes	No	2009	2018	N/A	No	N/A	ND	ND	N/A
C	S	Private	IIV-egg	Seasonal	Bilateral	Fill/finish	Yes	Yes	2018	2023	2023	Yes	ND	ND	ND	ND
O	R	Public	IIV-cell	Seasonal	Bilateral	Large-scale	ND	N/A	ND	N/A	N/A	NA	N/A	N/A	N/A	N/A
T	R	Other	IIV-egg	Seasonal	Multilateral	Fill/finish	No	N/A	2011	2016	N/A	N/A	N/A	N/A	N/A	N/A
T	R	Private	RIV-cell	Seasonal	Bilateral	Large-scale	No	N/A	2016	2019	N/A	N/A	N/A	N/A	N/A	N/A

[1] C: completed, O: ongoing (as of 2023), T: terminated

[2] R: recipient, S: supplier

[3] N/A: Not applicable

[4] ND: No data provided by participant

S8. Summary table of importance rating for TT enablers

Enabler	n	Median	Mean (95% CI)	Standard deviation	Minimum	Maximum	Interquartile range
Access to appropriate capital markets (i.e., project funding)	24	4	4.04 (3.58-4.50)	1.08	1	5	2
Adherence to ethical standards	24	5	4.25 (3.75-4.75)	1.19	1	5	1
Adherence to regulatory standards	24	5	4.92 (4.80-5.04)	0.28	4	5	0
Business development experience	24	4	4.17 (3.78-4.55)	0.92	2	5	1
Clear economic development priorities	24	4	4.21 (3.96-4.46)	0.59	3	5	1
Common values, trust, and commitment from all parties	24	5	4.54 (4.29-4.79)	0.59	3	5	1
Experience with evaluating the quality of technologies	24	4.5	4.33 (4.01-4.65)	0.76	3	5	1
Functional NRA	24	5	4.63 (4.38-4.87)	0.58	3	5	1
Government commitment to local production/procurement of vaccine	24	5	4.54 (4.24-4.85)	0.72	3	5	1
Innovation-friendly environment with sound IP rights	23	4	3.91 (3.52-4.30)	0.90	2	5	2
Political stability and transparent governance	24	4	3.83 (3.49-4.18)	0.82	2	5	1
Proper access to information (e.g., available resources, market opportunities)	24	4	3.97 (3.70-4.23)	0.63	3	5	0
R&D capacity of the technology recipient	24	4	4.00 (3.57-4.43)	1.02	2	5	2
Resource prioritization at the company level	24	4	4.33 (4.13-4.54)	0.48	4	5	1
Rule of Law/legal system is established and enforced	24	4	4.00 (3.69-4.31)	0.72	3	5	2
Viable and accessible local market	24	5	4.50 (4.17-4.83)	0.78	2	5	1
Workforce recruitment, training, retention	24	5	4.54 (4.33-4.76)	0.51	4	5	1

S9. Summary table of weighted ranks for TT enablers

Enabler	n	Median	Mean (95% CI)	Standard deviation	Minimum	Maximum	Interquartile range	Sum
Access to appropriate capital markets (i.e., project funding)	24	0	1.17 (0.44-1.89)	1.71	0	5	3	28
Adherence to ethical standards	24	0	1.21 (0.30-2.11)	2.15	0	5	3	29
Adherence to regulatory standards	24	2	2.08 (1.28-2.89)	1.91	0	5	4	50
Business development experience	24	0	0.38 (-0.05-0.80)	1.01	0	4	0	9
Clear economic development priorities	24	0	0.17 (-0.04-0.37)	0.48	0	2	0	4
Common values, trust, and commitment from all parties	24	0	1.08 (0.33-1.84)	1.79	0	5	3	26
Experience with evaluating the quality of technologies	24	0	0.46 (0.06-0.85)	0.93	0	4	1	11
Functional NRA	24	0	1.21 (0.54-1.88)	1.59	0	5	3	29
Government commitment to local production/procurement of vaccine	24	2.5	2.54 (1.68-3.40)	2.04	0	5	5	61
Innovation-friendly environment with sound IP rights	24	0	0.13 (-0.13-0.38)	0.61	0	3	0	3
Political stability and transparent governance	24	0	0.29 (-0.17-0.75)	1.08	0	5	0	7
Proper access to information (e.g., available resources, market opportunities)	24	0	0.04 (-0.04-0.13)	0.20	0	1	0	1
R&D capacity of the technology recipient	24	0	0.50 (-0.03-1.03)	1.25	0	4	0	12
Resource prioritization at the company level	24	0	0.63 (0.06-1.19)	1.35	0	5	0	15
Rule of Law/legal system is established and enforced	24	0	0.21 (-0.10-0.51)	0.72	0	3	0	5
Viable and accessible local market	24	2	1.92 (1.10-2.73)	1.93	0	5	4	46
Workforce recruitment, training, retention	24	0.5	1.00 (0.42-1.58)	1.38	0	5	2	24

S.10 Additional IVTT recipients and supplier

Institution, Country	Determinants	Effectiveness criteria	Enablers/barriers	References
Recipients				
Birmex, Mexico	• TR: Public manufacturer; Prior experience with production of other vaccines<br>• TS: Sanofi; WHO TTI<br>• TO: Egg-based IIV<br>• TM: Bilateral agreement (2007) – fill/finish; Multilateral agreement (joined WHO TTI in 2007) – complementary to bilateral agreement<br>• DE: Seasonal influenza vaccines introduced in 2004 and target groups established; National pandemic preparedness plan called for domestic production of influenza vac-cines in 2007; MOH established 15-year advanced purchase agreement with Birmex in 2008; NRA not functional prior to IVTT	• OTD: IVTT completed<br>• MI: A(H1N1) pandemic influenza vaccine approved in 2009; Status of seasonal influenza vaccine packaged by Birmex is un-known<br>• HC: Staff trained through WHO-facilitated course on quality control (QC), Sanofi workshops on production methods and engineering, and North Carolina State University courses on influenza vaccine manufacturing<br>• PV: Fill/finish capacity reported as 30 mil-lion doses for seasonal influenza vaccines and up to 60 million doses for pandemic influenza vaccines	• Preferential procurement of domestic products even if they're up to 15% higher than other products<br>• TT agreement with Sanofi included supply assurance of 15 million doses of seasonal vaccines each year<br>• TT agreement with Sanofi limited export without prior approval<br>• Multi-year agreement with the MOH set the maximum price equal to vaccines offered through the PAHO Revolving Fund but procurement of the bulk antigen from Sanofi influenced the minimum price it could be sold<br>• Emphasis on seasonal influenza vaccination in national pandemic preparedness plan<br>• Access to capital for fill/finish facility and equipment enabled by WHO TTI grants and national funding<br>• WHO regulatory capacity-building efforts led to functional NRA status	[1-6]
Cantacuzino Institute, Romania	• TR: Public manufacturer; Prior experience with production of influenza and other vaccines<br>• TS: WHO TTI; Infectious Disease Research Institute for adjuvant<br>• TO: Egg-based IIV (seasonal); oil-in-water adjuvant	• OTD: IVTT completed<br>• MI: A(H1N1) pandemic vaccine approved in 2011<br>• HC: Staff trained through Utah State University and North Carolina State University courses on influenza vaccine manufacturing	• Political instability led to change in management structure and financial insecurity<br>• Access to capital through WHO grants enabled preclinical and clinical studies<br>• Public was reluctant to accept A(H1N1) pandemic vaccine due to poor marketing	[5-11]

	• TM: Multilateral agreement (joined WHO TTI in 2009) – pilot stage production; Bilateral agreement for adjuvant<br>• DE: Motivation was to optimize influenza vaccine production; Seasonal influenza vaccines used in Romania prior to IVTT; NRA not functional prior to IVTT		• Ceased commercial operations after regulator withdrew the institute's authorization to produce vaccines in 2010 due to regulatory compliance issues and failure to produce influenza vaccines in 2013<br>• Workforce was reduced due to state budget limitations, influenza staff reduced below lower limit needed for production	
Mechnikov Institute, Nicaragua	• TR: Private manufacturer; No prior experience in TT or production of influenza or other vaccines<br>• TS: St. Petersburg Scientific Research Institute of Vaccines and Sera<br>• TO: Egg-based IIV (seasonal)<br>• TM: Bilateral agreement (IVTT initiated in 2015) – stepwise transfer of fill/finish capacity followed by full-scale production<br>• DE: Seasonal influenza vaccines used in Nicaragua prior to IVTT; Motivation to supply to the Pan American Health Organization (PAHO) Revolving Fund; NRA not functional prior to IVTT	• OTD: IVTT completed<br>• MI: Seasonal influenza vaccine approved in 2019 and achieved WHO PQ in 2025<br>• OC: Received COVID-19 vaccine production technology from the same supplier<br>• PV: Production capacity of 30 million doses of seasonal influenza vaccines each season; Ability to produce NH and SH vaccine formulations	• Establishment of facility and IVTT supported by Government of Nicaragua and Russian Federation Ministry of Health<br>• PAHO support for NRA capacity-building efforts	[12-15]
Razi Institute, Islamic Republic of Iran	• TR: Public manufacturer; Prior experience producing human and veterinary vaccines, including a veterinary influenza vaccine<br>• TS: WHO TTI<br>• TO: Egg-based IIV<br>• TM: Multilateral agreement (joined WHO TTI in 2009) – full scale production	• OTD: IVTT terminated before completion although A(H1N1) pandemic vaccines were produced at lab scale<br>• HC: Staff trained through NVI courses on quality assurance, QC, and influenza vaccine manufacturing	• Access to centrifuges necessary for whole-virion IIV production cited as a barrier, leading to review of LAIV as a better option<br>• WHO regulatory capacity-building efforts led to functional NRA status	[1,2,5,8]

	• DE: Motivation for full production facility; NRA not functional prior to IVTT			
Research Institute for Biological Safety and Problems, Kazakhstan	• TR: Public institute; Prior experience with R&D of A(H5N1) and A(H1N1) monovalent influenza vaccines and veterinary influenza vaccines<br>• TS: WHO TTI<br>• TO: Egg-based IIV (seasonal)<br>• TM: Multilateral agreement (joined WHO TTI in 2011)<br>• DE: Motivation to produce six million doses of seasonal influenza vaccines; NRA not functional prior to IVTT	• OTD: IVTT completed<br>• MI: Seasonal influenza vaccine approval unknown<br>• PA: Funding for clinical trials of seasonal IIV provided by government<br>• HC: Staff trained through North Carolina State University courses on influenza vaccine manufacturing	• Access to capital through WHO grants focused on optimization methods was an enabler<br>• Lack of access to capital for a production facility was a barrier	[5,8,16-18]
Serum Institute of India, India	• TR: Private manufacturer; Prior experience with vaccine production and TT; Prior experience with egg- and cell-based IIV R&D<br>• TS: WHO TTI, BioDiem, and Nobilon<br>• TO: Egg-based IIV and LAIV (seasonal and pandemic)<br>• TM: Multilateral agreement (joined WHO TTI in 2007) – LAIV sublicense obtained in 2009<br>• DE: No national influenza vaccination policy prior to IVTT; Entered into advanced market commitment with the government in 2009 for A(H1N1) pandemic vaccine supply; Functional NRA prior to IVTT	• OTD: IVTT completed<br>• MI: A(H1N1) pandemic LAIV and IIV approved in 2010; A(H1N1) pandemic LAIV achieved WHO PQ in 2012; Seasonal LAIV approved in 2014 and achieved WHO PQ in 2015<br>• PA: Government funding followed WHO TTI participation<br>• HC: Staff trained through NVI courses on laboratory and QC techniques and production processes and through North Carolina State University courses on influenza vaccine manufacturing<br>• OC: Explored cell-based LAIV; Post-licensure safety and efficacy studies of LAIV conducted in Bangladesh, Senegal, and Gambia<br>• PV: Pandemic production capacity reported to be at least 200 million doses	• Limited influenza vaccine demand and lack of seasonal influenza vaccination policy in India noted as barriers to market impact<br>• Government supported noted as an enabler for A(H1N1) pandemic vaccine development but lacked support for sustaining seasonal vaccine production<br>• IP barriers for use of reverse genetics in LAIV development<br>• Sourcing of eggs and vaccine viruses identified as a barrier	[1,6-8,16,19-23]

VABIOTECH, Viet Nam	• TR: Public manufacturer; Prior experience with vaccine production<br>• TS: PATH<br>• TO: Cell-based IIV (pandemic)<br>• TM: Bilateral agreement – R&D and process development<br>• DE: NRA not functional prior to IVTT	• OTD: IVTT status unknown<br>• HC: Staff trained through North Carolina State University courses on influenza vaccine manufacturing	• Access to license for cell lines necessary for influenza vaccine manufacturing facilitated by PATH<br>• Access to capital through US government grants was an enabler	[5,6,19,24]
Vacsera, Egypt	• TR: Public manufacturer; Prior experience with vaccine production<br>• TS: WHO TTI<br>• TO: Egg-based IIV<br>• TM: Multilateral agreement (joined WHO TTI in 2009); Bilateral agreement with NVI for specific training needs<br>• DE: NRA not functional prior to IVTT	• OTD: IVTT terminated<br>• HC: Staff trained through NVI courses on laboratory and QC techniques and production processes and through Utah State University and North Carolina State University courses on influenza vaccine manufacturing<br>• OC: Reported to produce influenza vaccines through IVTT with GC Biopharma	• WHO regulatory capacity-building efforts led to functional NRA status<br>• Access to capital through WHO grants for equipment, training, and preclinical/clinical studies was an enabler	[1,6,8,11,25,26]
Supplier				
Netherlands Vaccine Institute, Netherlands	• TR: Multiple public and private manufacturers<br>• TS: Public institute; Prior experience with production and TT of other vaccines<br>• TO: Egg-based IIV (seasonal and pandemic)<br>• TM: Multilateral agreement (selected as IVTT hub for WHO TTI in 2007) – pilot stage production; Bilateral agreements for individual trainings<br>• DE: NVI selected as hub due to manufacturing and QC experience and financial support	• OTD: IVTT completed<br>• MI: Multiple seasonal and pandemic influenza vaccine approvals by manufacturers trained at hub<br>• HC: At least 65 participants from 18 institutes received training in influenza vaccine production and QC; Onsite training provided to two manufacturers	• Simplicity, immunogenicity, and robustness of egg-based whole virion IIV production processes and lack of IP barriers noted as enablers for IVTT<br>• Technology assessment led to addition of split virion production process to the curriculum to support seasonal IIV program development<br>• Trainings also included participants from NRAs<br>• Demand for practical trainings declined following the 2009 A(H1N1) pandemic	[8,25,27-29]

Abbreviations: DE: demand environment, HC: human capital, MI: market impact, OC: opportunity costs, OTD: out-the-door, PA: political advantage, PV: public value, TO: transfer object, TM: transfer media, TR: transfer recipient, TS: transfer supplier

S11. References for supplementary materials

1. Chadwick, C.; Friede, M.; Moen, A.; Nannei, C.; Sparrow, E. Technology transfer programme for influenza vaccines–Lessons from the past to inform the future. *Vaccine* **2022**, *40*, 4673, doi:10.1016/j.vaccine.2022.06.057.
2. World Health Organization. Report of the fourth meeting with international partners on prospects for influenza vaccine technology transfer to developing country vaccine manufacturers, Cancun, Mexico, 4-5 May 2011. Available online: https://iris.who.int/bitstream/handle/10665/44750/9789241502597_eng.pdf (accessed on 5 January 2026).
3. Ponce-de-Leon, S.; Velazquez-Fernandez, R.; Bugarin-González, J.; García-Bañuelos, P.; Lopez-Sotelo, A.; Jimenez-Corona, M.-E.; Padilla-Catalan, F.; Cervantes-Rosales, R. Domestic influenza vaccine production in Mexico: a state-owned and a multinational company working together for public health. *Vaccine* **2011**, *29*, A26–A28.
4. World Health Organization. *Technical Report of consultations with the Mexican Secretary of Health on key elements of sustainability for local production of influenza vaccine within the context of global pandemic preparedness*; World Health Organization: 2016.
5. Palkonyay, L.; Fatima, H. A decade of adaptation: Regulatory contributions of the World Health Organization to the Global Action Plan for Influenza Vaccines (2006–2016). *Vaccine* **2016**, *34*, 5414–5419, doi:10.1016/j.vaccine.2016.07.025.
6. Ruiz, J.; Gilleskie, G.L.; Brown, P.; Burnett, B.; Carbonell, R.G. Comprehensive hands-on training for influenza vaccine manufacturing: A WHO–BARDA–BTEC partnership for global workforce development. *Biochemistry and Molecular Biology Education* **2014**, *42*, 414–419, doi:10.1002/bmb.20817.
7. Grohmann, G.; Francis, D.P.; Sokhey, J.; Robertson, J. Challenges and successes for the grantees and the Technical Advisory Group of WHO's influenza vaccine technology transfer initiative. *Vaccine* **2016**, *34*, 5420–5424, doi:10.1016/j.vaccine.2016.07.047.
8. World Health Organization. Meeting with international partners on influenza vaccine production technology transfer to developing countries, 5-6 May 2010, Nha Trang, Viet Nam. Available online: https://iris.who.int/bitstream/handle/10665/70502/WHO_IVB_10.10_eng.pdf?sequence=1 (accessed on 5 January 2026).
9. Stavaru, C.; Onu, A.; Lupulescu, E.; Tucureanu, C.; Rasid, O.; Vlase, E.; Coman, C.; Caras, I.; Ghiorghisor, A.; Berbecila, L. Technology transfer of oil-in-water emulsion adjuvant manufacturing for pandemic influenza vaccine production in Romania: Preclinical evaluation of split virion inactivated H5N1 vaccine with adjuvant. *Human vaccines & immunotherapeutics* **2016**, *12*, 1009–1026.
10. Blume, S.; Baylac-Paouly, B. *Immunization and states: the politics of making vaccines*; Routledge: 2021.
11. Tarbet, E.B.; Dorward, J.T.; Day, C.W.; Rashid, K.A. Vaccine production training to develop the workforce of foreign institutions supported by the BARDA influenza vaccine capacity building program. *Vaccine* **2013**, *31*, 1646–1649, doi:10.1016/j.vaccine.2012.06.041.
12. Latin-American Institute of Biotechnology Mechnikov. Available online: https://mechnikov.com/en/main (accessed on 5 January 2026).
13. World Health Organization. Report of the eighth meeting with international partners on prospects for influenza vaccine technology transfer to developing country vaccine manufacturers: Sao Paulo, Brazil, 17–18 March 2015. Available online: https://iris.who.int/bitstreams/7cac40ac-df24-4053-8af7-e2ae0e29e4b9/download (accessed on 5 January 2026).
14. World Health Organization. WHO/UNICEF Joint Reporting Form on immunization: Influenza vaccination policy. Available online: https://immunizationdata.who.int/global/wiise-detail-page/influenza-vaccination-policy (accessed on 5 January 2026).
15. World Health Organization. WHO Public Inspection Report: Instituto Latinoamericano de Biotecnologia Mechnikov SA. Available online:

https://extranet.who.int/prequal/sites/default/files/whopir_files/WHOPIR_Mechnikov_24-28April2023.pdf (accessed on 5 January 2026).
16. World Health Organization. Report of the fifth meeting with international partners on prospects for influenza vaccine technology transfer to developing country vaccine manufacturers, Belgrade, Serbia, 27-28 March 2012. Available online: https://iris.who.int/bitstream/handle/10665/44869/9789241503518_eng.pdf (accessed on 5 January 2026).
17. Sarsenbayeva, G.; Volgin, Y.; Kassenov, M.; Issagulov, T.; Bogdanov, N.; Sansyzbay, A.; Stukova, M.; Buzitskaya, Z.; Kulmagambetov, I.; Davlyatshin, T.; et al. Immunogenicity and safety of a novel seasonal influenza preservative-free vaccine manufactured in Kazakhstan: Results of a randomized, comparative, phase II clinical trial in adults. *Human Vaccines & Immunotherapeutics* **2018**, *14*, 609–614, doi:10.1080/21645515.2017.1387345.
18. Sarsenbayeva, G.; Issagulov, T.; Kassenov, M.; Abitay, R.; Orynbayev, M.; Stukova, M.; Pisareva, M.; Davlyatshin, T.; Lespek, K.; Khairullin, B. Safety and immunogenicity of trivalent inactivated influenza vaccine in adults 60 years of age and older: a phase II, a randomized, comparative trial in Kazakhstan. *Human Vaccines & Immunotherapeutics* **2020**, *16*, 1791–1797, doi:10.1080/21645515.2019.1705691.
19. Perdue, M.L.; Bright, R.A. United States of America Department of Health and Human Services support for advancing influenza vaccine manufacturing in the developing world. *Vaccine* **2011**, *29*, A48–A50, doi:10.1016/j.vaccine.2011.02.080.
20. Jadhav, S.; Dhere, R.; Yeolekar, L.; Gautam, M. Influenza Vaccine Production Capacity Building in Developing Countries: Example of the Serum Institute of India. *Procedia in Vaccinology* **2010**, *2*, 166–171, doi:https://doi.org/10.1016/j.provac.2010.07.010.
21. Dhere, R.; Yeolekar, L.; Kulkarni, P.; Menon, R.; Vaidya, V.; Ganguly, M.; Tyagi, P.; Barde, P.; Jadhav, S. A pandemic influenza vaccine in India: From strain to sale within 12 months. *Vaccine* **2011**, *29*, A16–A21, doi:https://doi.org/10.1016/j.vaccine.2011.04.119.
22. World Health Organization. Report of the ninth meeting with international partners on prospects for influenza vaccine technology transfer to developing country vaccine manufacturers: Geneva, Switzerland, 17 November 2016. Available online: https://iris.who.int/bitstreams/f4cd2ff5-5add-43f6-b318-e8e06dd72fe3/download (accessed on 5 January 2026).
23. Adbi, A.; Chatterjee, C.; Drev, M.; Mishra, A. When the Big One Came: A Natural Experiment on Demand Shock and Market Structure in India's Influenza Vaccine Markets. *Production and Operations Management* **2019**, *28*, 810–832, doi:https://doi.org/10.1111/poms.12948.
24. Scorza, F.B. Advancing new vaccines against pandemic influenza in low-resource countries. *Vaccine* **2017**, *35*, 5397–5402, doi:10.1016/j.vaccine.2017.03.094.
25. World Health Organization. Meeting with international partners on prospects for influenza vaccine technology transfer to developing countries, 27-28 November 2008, Pune, Maharashtra, India. Available online: https://iris.who.int/bitstream/handle/10665/70016/WHO_IVB_09.06_eng.pdf (accessed on 5 January 2026).
26. Egypt National Strategy for Vaccine Manufacturing Localization 2024–2030. Available online: https://upa.gov.eg/wp-content/uploads/2025/06/National_Vaccine_Manufacturing_Localization_Strategy.pdf (accessed on 5 January 2026).
27. Hendriks, J.; Holleman, M.; de Boer, O.; de Jong, P.; Luytjes, W. An international technology platform for influenza vaccines. *Vaccine* **2011**, *29*, A8–A11, doi:10.1016/j.vaccine.2011.04.124.
28. Friede, M.; Serdobova, I.; Palkonyay, L.; Kieny, M.P. Technology transfer hub for pandemic influenza vaccine. *Vaccine* **2009**, *27*, 631–632, doi:https://doi.org/10.1016/j.vaccine.2008.10.080.
29. Hendriks, J.; Holleman, M.; Hamidi, A.; Beurret, M.; Boog, C. Vaccinology capacity building in Europe for innovative platforms serving emerging markets. *Human Vaccines & Immunotherapeutics* **2013**, *9*, 932–936, doi:10.4161/hv.23163.
